# Treatment of Refractory Cardiac Arrest by Controlled Reperfusion of the Whole Body: A Multicenter, Prospective Observational Study

**DOI:** 10.3390/jcm13010056

**Published:** 2023-12-21

**Authors:** Georg Trummer, Christoph Benk, Jan-Steffen Pooth, Tobias Wengenmayer, Alexander Supady, Dawid L. Staudacher, Domagoj Damjanovic, Dirk Lunz, Clemens Wiest, Hug Aubin, Artur Lichtenberg, Martin W. Dünser, Johannes Szasz, Dinis Dos Reis Miranda, Robert J. van Thiel, Jan Gummert, Thomas Kirschning, Eike Tigges, Stephan Willems, Friedhelm Beyersdorf, on behalf of the Extracorporeal Multi-Organ Repair Study Group

**Affiliations:** 1 Department of Cardiovascular Surgery, University Medical Center Freiburg, University of Freiburg, Hugstetter Str. 55, 79106 Freiburg, Germany; georg.trummer@uniklinik-freiburg.de (G.T.);; 2 Faculty of Medicine, Albert-Ludwigs-University Freiburg, Breisacherstr. 153, 79110 Freiburg, Germany; 3 Department of Emergency Medicine, Medical Center—University of Freiburg, Hugstetter Str. 55, 79106 Freiburg, Germany; 4 Interdisciplinary Medical Intensive Care, Medical Center—University of Freiburg, 79106 Freiburg, Germany; 5 Department of Anesthesiology, University Medical Center, 93042 Regensburg, Germany; dirk.lunz@ukr.de; 6 Department of Internal Medicine II, University Medical Center, 93042 Regensburg, Germany; 7 Department of Cardiac Surgery, Medical Faculty and University Hospital Düsseldorf, Heinrich-Heine-University Düsseldorf, 40225 Düsseldorf, Germanyartur.lichtenberg@med.uni-duesseldorf.de (A.L.); 8 Department of Anesthesiology and Intensive Care Medicine, Kepler University Hospital and Johannes Kepler University, 4020 Linz, Austria; 9 Department of Adult Intensive Care, Erasmus MC University Medical Center, 3015 GD Rotterdam, The Netherlands; 10 Clinic for Thoracic and Cardiovascular Surgery, Heart and Diabetes Center NRW, University Hospital of the Ruhr University Bochum, 44791 Bad Oeynhausen, Germany; 11 Asklepios Klinik St. Georg, Heart and Vascular Center, Department of Cardiology and Intensive Care Medicine, 20099 Hamburg, Germany

**Keywords:** cardiac arrest, cardiopulmonary resuscitation, organ repair, extracorporeal circulation, extracorporeal cardiopulmonary resuscitation

## Abstract

*Background:* Survival following cardiac arrest (CA) remains poor after conventional cardiopulmonary resuscitation (CCPR) (6–26%), and the outcomes after extracorporeal cardiopulmonary resuscitation (ECPR) are often inconsistent. Poor survival is a consequence of CA, low-flow states during CCPR, multi-organ injury, insufficient monitoring, and delayed treatment of the causative condition. We developed a new strategy to address these issues. *Methods:* This all-comers, multicenter, prospective observational study (69 patients with in- and out-of-hospital CA (IHCA and OHCA) after prolonged refractory CCPR) focused on extracorporeal cardiopulmonary support, comprehensive monitoring, multi-organ repair, and the potential for out-of-hospital cannulation and treatment. *Result:* The overall survival rate at hospital discharge was 42.0%, and a favorable neurological outcome (CPC 1+2) at 90 days was achieved for 79.3% of survivors (CPC 1+2 survival 33%). IHCA survival was very favorable (51.7%), as was CPC 1+2 survival at 90 days (41%). Survival of OHCA patients was 35% and CPC 1+2 survival at 90 days was 28%. The subgroup of OHCA patients with pre-hospital cannulation showed a superior survival rate of 57.1%. *Conclusions:* This new strategy focusing on repairing damage to multiple organs appears to improve outcomes after CA, and these findings should provide a sound basis for further research in this area.

## 1. Introduction

Sudden cardiac arrest (CA) is one of the leading causes of mortality worldwide, with cardiac events being the predominant cause. Survival and neurological recovery after more than 20–30 min of conventional cardiopulmonary resuscitation (CCPR) remain dismal, even with the use of advanced life-support techniques (ALS) [1,2,3]. Current survival for out-of-hospital cardiac arrest (OHCA) is <10% worldwide [4] (ranging from 6 to 20% [5,6,7,8,9]) and in-hospital cardiac arrest (IHCA) is <14% [10] (ranging from 6 to 26% [3,10,11,12,13]). A substantial number of surviving patients suffer from significant neurological dysfunction [1,14,15].

Extracorporeal cardiopulmonary resuscitation (ECPR) was introduced more than 15 years ago [16,17] in order to improve survival. Many ECPR registry studies as well as three recent randomized trials have been published, with varying results.

In an attempt to further improve the results after prolonged refractory CA, our group has developed next-generation ECPR technology we have named “controlled reperfusion of the whole body (CARL)”. This innovative strategy applies a multimodal therapy that addresses the pathophysiology of injuries induced by CA and prolonged CCPR. Our concept consists of four main components (described in detail in the Supplementary Materials, Tables S2 and S7, Box S1):

1. Extracorporeal circulation: As opposed to regular extracorporeal circuits, CARL contains dual diagonal pumps for pulsatility and high pressure and flow (Supplementary Materials, Figure S1) as well as controlled oxygenation to limit free radical injury. It induces immediate (<20 min) systemic mild hypothermia (33–35 °C).
2. Individualized multi-organ repair: Instead of returning the usually hyperoxemic, but otherwise unmodified, blood into the femoral artery, the system adjusts 14 blood parameters (e.g., hypocalcemia, hypermagnesemia, hyperosmolarity, low oxygen, free radical scavengers, etc.) [18] to counteract ischemic/hypoxic and reperfusion/reoxygenation injury to diverse organ systems. These adjustments can be made individually for each patient using the data obtained from the real-time monitoring.
3. Comprehensive real-time monitoring: This allows for real-time measurements of hemodynamic (cardiac output, heart rate, and blood pressure), metabolic (blood gases and electrolytes), and temperature parameters and provides personalized treatment by an immediate adjustment to the desired value of each parameter.
4. Option for out-of-hospital CARL treatment using suitable mobile devices: This allows pre-hospital cannulation and the early start of CARL in order to reduce the duration of CCPR.

We experimentally developed this concept [18,19,20], and the principle has been confirmed by other groups [19,21,22]. Clinical application has recently begun, and case reports [23,24] and a case series [25] have been published. This is the first clinical multicenter prospective observational study with preliminary results using the new CARL treatment for prolonged refractory CA. This observational study differs from previous ECPR trials with regards to various aspects (no randomization, no control group, an all-comers study, inclusion of IHCA and OHCA, and no pre-defined inclusion or exclusion criteria) because an all-comers design was chosen. This was due to the unpredictability of determining which subgroups of patients might benefit from this new treatment. As we did not have a control group in this first report, our data are presented within the framework of historical ECPR data from our own institution and previously published registry and randomized studies.

The objectives of this first prospective observational multicenter study with an all-comers cohort were to (1) test the hypothesis that improvements in the outcomes after prolonged refractory CA in various subgroups might be achieved by applying the concept of controlled automated reperfusion of the whole body, and (2) evaluate the quality of organ functions, including the brain, myocardium, kidney, and liver.

## 2. Materials and Methods

### 2.1. Clinical Study, Design, and Statistical Analysis Plan

This controlled automated reperfusion of the whole body (CARL) study (phase I/II clinical trial) was a multicenter, international, prospective non-interventional, open, and single-arm study. To assess the heterogeneity of resuscitated patients, data were reported according to the Utstein style [26,27].

### 2.2. Study Setting

A total of 69 patients with CA were enrolled from January 2020 to January 2023 at 7 centers across 3 European countries (Freiburg-Bad Krozingen, Regensburg, Düsseldorf, Linz, Rotterdam, Bad Oeynhausen, and Hamburg). All centers were highly experienced in ECMO application for circulatory (VA ECMO) or respiratory (VV ECMO) therapy before the start of this trial. Ethics committee approval was obtained from the University of Freiburg (Germany) (120/19 (MPG §23b, 5 September 2019) and this approval was confirmed thereafter by all ethics committees of the participating centers. Prior to study initiation, emergency medical services (EMS) and hospital staff were trained to deliver the CARL therapy according to a study protocol plan (Supplementary Materials, Box S2 and Figure S2) with some center-specific modifications. Due to the COVID-19 pandemic, enrollment was prolonged. Follow-up assessments of all parameters were conducted until hospital discharge and achieved a completion rate of 100%. In addition, a follow-up of the neurological status of the surviving patients discharged from the hospital with CPC 3 (n = 13) was performed up to 90 and 180 days, with a follow-up rate of 100%.

### 2.3. Patients

The 69 patients initially included in this study had refractory in- or out-of-hospital CA (Table 1). Patients with cardiogenic shock or post-cardiotomy or respiratory failures were not included. As this was an observational study using a CE-certified device, informed consent had to be given only for data processing and not for the treatment with the CARL Controller. Treatment with the CE-mark-approved system could be performed in routine clinical practice, and the decision to use extracorporeal multi-organ repair was made by the treating physician according to the patient’s presumed will for resuscitation. Patients were generally treated for at least several days and up to many weeks in an intensive care unit. During the observational period, informed consent for data processing was retrospectively obtained either from the patient or from their legal representative. All data processing was conducted according to the current European General Data Protection Regulation (GDPR) guidelines. Further information about the patients can be found in the Supplementary Materials (Methods).

### 2.4. Primary and Secondary Endpoints

The primary endpoints were defined as overall survival at hospital discharge and a good neurological outcome (CPC score 1 or 2) at hospital discharge [28]. The CPC scale ranges from 1 (good cerebral performance) to 5 (brain death). A favorable CPC score is defined as ≤2. The clinical effectiveness endpoints were the number of patients who survived and the number of patients who survived with a good neurological outcome. The list and definitions of secondary endpoints can be found in the Supplementary Materials (Methods).

### 2.5. Procedure

The concept of controlled automated reperfusion of the whole body (CARL) was implemented using the CARL Controller^®^ (the main technical component for multi-organ repair, high pulsatile pressure and flow, and comprehensive monitoring), the CARL MOX^®^ (used as an oxygen provider), and the CARL Cooler^®^ (for immediate cooling of the patient) (Resuscitec GmbH, Freiburg, Germany).

The patients involved in this study were percutaneously cannulated using the Seldinger technique, with the guidance of ultrasound employed in some centers. The cannulation process involved the use of conventional femoral cannulas for arterial and venous cannulation. To prevent leg ischemia, some centers employed antegrade leg perfusion using a wire-braided 8Fr vascular cannula (free life medical GmbH, Aachen, Germany). As part of the cannulation procedure, the cannulas were immediately flushed at most centers with 100 mL normal saline solution and 5000 IU heparin after implantation.

**Figure 1:**
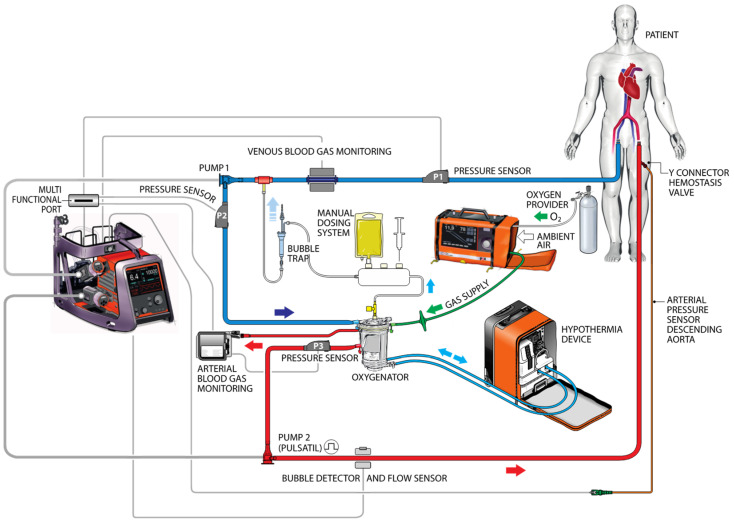
Schematic presentation of the controlled automated reperfusion of the whole body and overview of the technology.

Following patient cannulation, the pre-prepared CARL Controller^®^ (Resuscitec), which includes a reperfusion set with two diagonal high-performance pump systems, an oxygenator, sensors, a drug-delivery system, a venting line, monitoring devices, and an automated priming mode (Figure 1), was connected to the cannula. Real-time monitoring of hemodynamic parameters (cardiac output, heart rate, and blood pressure), venous and arterial blood gases (pO_2_ and pH), electrolytes (calcium, potassium, and sodium), and venous blood temperature was implemented to achieve the necessary personalized treatment for multi-organ repair. Further details of the different strategies pertaining to the use of CCPR, ECPR, and CARL are shown in the Supplementary Materials, Table S7 and Box S1. The rationale behind the composition of the reperfusate was described in a previous publication [20].

### 2.6. Patients with “Non-Survivable” CA

The EMS classified the CA of 9 patients as “non-survivable”, based on pathoanatomical findings (n = 2 with ventricular rupture after acute MI; n = 7 with acute type A dissection, aortic rupture, and pericardial tamponade with subsequent CA). Further details are described in the Supplementary Materials, Box S3.

### 2.7. Statistical Analysis

Data collection was performed using dedicated case-report forms that complied with the applicable data protection requirements at the respective centers. Categorical variables were reported as percentages. Continuous variables were reported as means with standard deviations (if normally distributed) or as medians with interquartile ranges (IQRs). The Shapiro–Wilk test was used to test continuous variables for normal distribution. Spearman’s rank correlation coefficient (ρ) was calculated to analyze associations between variables. ρ values of 0.10, 0.30, and 0.50 were generally interpreted as small, moderate, and large effects, respectively. Two-sided *p*-values were provided. Further statistical information is given in the Supplementary Materials (Methods).

## 3. Results

### 3.1. Baseline Demographics

The mean patient age of our cohort was 59.0 ± 13.4 years, with 33% being older than 65 years. The group was predominantly male (78%) and the median body weight was 84 kg (interquartile range (IQR) 80–96 kg); 80% of the patients had known cardiovascular conditions and 55% had other co-morbidities, very often in combination (Table 1). The cause of CA was myocardial infarction in 62% (including 4.7% with free ventricular rupture), aortic type A dissection with subsequent CA in 10.1%, and other cardiovascular causes in 17%.

### 3.2. Pre-Hospital Resuscitation Characteristics

OHCA occurred in 58% of the patients (Table 1), and 35% of these (14/40) were cannulated outside of the hospital (Table 2). Eight patients with unwitnessed CA (12%) were also included in the study. The initial rhythm monitored at CA was shockable (ventricular fibrillation or pulseless ventricular tachycardia (PVT)) in only 53% and non-shockable (asystole or pulseless electrical activity (PEA)) in 47%. The severity of both no- and low-flow time (CA and CCPR) was reflected by the mean pH (7.0 ± 0.3) and median lactate (10.1 (IQR 7.7–12.3) mmol/L) values just before the start of extracorporeal circulation. The relatively favorable values for the median end-tidal CO_2_ (EtCO2) of 25 mmHg (IQR 20–30 mmHg; measured only in 11 of 69 patients) reflected the fact that CCPR was efficiently performed, even though CA remained refractory. Of note, 9 patients included had essentially “non-survivable” disease (Table 2) leading up to CA (for further details of these patients, see the Supplementary Materials).

### 3.3. Primary Outcome

Overall survival at hospital discharge was 42.0%. In the subgroup excluding patients with “non-survivable” causes of CA, the survival rate was 48.3% (Table 2). Overall, CPC 1 and 2 in surviving patients at 90 days was 79.3% (23/29); i.e., CPC 1 + 2 survival was 33% (Table 2). The most favorable survival outcomes were noted in the IHCA patients (51.7%) and in the OHCA patients who were cannulated out-of-hospital (57.1%).

We observed a correlation between the survival rate and both the age and duration of CCPR (Supplementary Table S6). A CCPR duration ≤ 30 min and an age range of 18–64 years were associated with 100% survival. Conversely, CCPR ≥ 60 min and an age ≥ 75 years were associated with 0% survival. However, patients aged 65–75 years demonstrated satisfactory survival rates across different CCPR durations (≤30 min (50%), 30–60 min (40%), and ≥60 min (29%)). Notably, all 4 patients aged > 75 years survived when the CCPR duration was ≤30 min.

The primary cause of death overall was either neurological (25%) or multi-organ failure (27.5%). The median duration of stay in the ICU was 8.1 days (IQR 1.5–23.0). Among the surviving patients, the median hospital stay was 28 days (IQR 16–49) (Table 2).

### 3.4. Time Intervals and Locations before, during, and after CA and CPR

**Figure 2:**
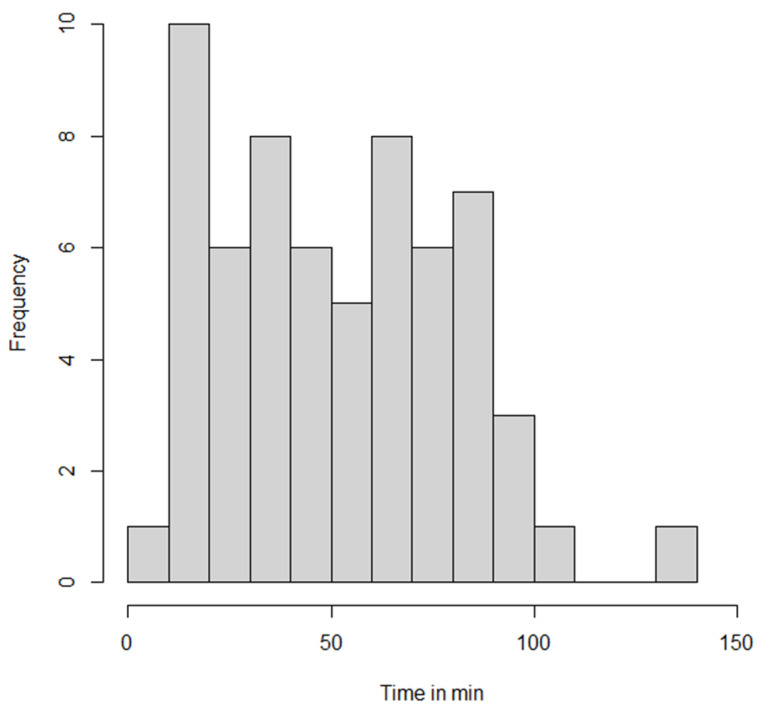
Duration of CCPR before the start of extracorporeal multi-organ therapy for N = 62/69 patients. Each column represents 10 min. The CCPR duration was >30 min for 73% of the patients; for 58% of the patients, the CCPR duration was >60 min.

In the OHCA patients, the duration between calling the emergency number and the arrival of the EMS team was 8.9 ± 4.9 min (Table 3). The duration of CCPR (low flow) for OHCA was 68.5 min (IQR 43.8–81.3) and for IHCA patients 33.5 min (IQR 18.5–49.3). CCPR duration was ≥30 min for 74% of all patients and >60 min for 55% (Figure 2). In the OHCA group (Table 3), 61% had a CCPR duration of ≥60 min. A significant difference was noted between the OHCA patients cannulated in the hospital (75 min; IQR 67.0–88.0; n = 25 needed transport from OHCA site to the hospital) and those cannulated pre-hospital (31.0 min; IQR 20.0–56.5; n = 11; *p* = 0.047). The time needed to establish extracorporeal circulation was ≤15 min for 56% and ≤20 min for 71%.

### 3.5. Organ Function Assessment

Among all the survivors at the point of hospital discharge, only one required renal replacement therapy and none required any form of mechanical circulatory support (Table 4). Only three patients, despite stable hemodynamics and intact neurological functions, required prolonged invasive ventilation. Over the course of the treatment, clinically relevant serum biomarkers for the kidney, liver, and respiratory or cardiovascular systems showed only a mild to moderate impairment. However, these elevated levels sufficiently recovered by the time of hospital discharge.

### 3.6. Clinical Events and Complications

Clinical events in patients who underwent extracorporeal circulation and CARL treatment are listed in the Supplementary Materials, Table S1. Bleeding was the most common complication, mostly related to coagulopathy and veno-arterial cannulation. Neurological events occurred, and it was difficult to distinguish between consequences of prolonged CA and CCPR and extracorporeal circulation. The rate of pseudosubarachnoid hemorrhage (pSAH) was 10% (Table S1) and did not result in any therapeutic consequences. Leg ischemia was documented in 8.7% and infection at all sites occurred in 11.6%. Further observed events were acute renal failure (13%), generalized inflammatory reactions (7%), and hemolysis (3%).

## 4. Discussion

This multicenter, prospective, all-comers observational study has provided preliminary data on a new approach to treat patients with prolonged refractory CA and CCPR by applying an advanced extracorporeal perfusion circuit, making efforts towards multi-organ repair, carrying out comprehensive monitoring, and offering the option for out-of-hospital initiation of treatment. As this was the first larger report about the clinical outcomes of this new approach, an all-comers study design was used to enroll a variety of patients, including IHCA and OHCA patients, shockable and non-shockable rhythms, prolonged CCPR duration up to >100 min, and all ages.

The overall survival rate at hospital discharge was 42.0%, and 79% of surviving patients demonstrated favorable neurological recovery at 90 days (CPC 1+2 survival at 90 days: 33%). Furthermore, severe, life-limiting, causative cardiac disease was observed in 13% of the patients. Overall survival for IHCA was 52% and survival with CPC 1+2 at 90 days was 41%. For OHCA, overall survival was 35% and CPC 1+2 survival at 90 days was 28%. Of note, overall survival of OHCA patients cannulated pre-hospital was 57% (Table 2). These encouraging outcomes were achieved despite a relatively older patient population, with 33% being older than 65 years, 12% having had an unwitnessed CA, and only 53% of patients with an initial rhythm amenable to defibrillation (ventricular fibrillation or pulseless ventricular tachycardia). The initial pH and lactate measurements were 7.0 and 10.3 mmol/L, respectively (Table 1). All patients had refractory CA and none of our patients had sustained spontaneous circulation just before extracorporeal circulation was started. CCPR lasted a median of 52 min (ranging up to 138 min) for all patients, while the duration for OHCA was >60 min for 61% (Table 3).

Current CCPR treatment after OHCA shows low survival rates (10–14%) for both OHCA [4,5,6,7,8,9,29,30,31] and IHCA [10]. These low CCPR survival rates arise from the delayed initiation of basic or advanced life support, the generation of a very low cardiac output (≤20–25%) from chest compressions [9,32], an inconsistent return of a robust cardiopulmonary function after CCPR, and complex pathophysiological and metabolic events after prolonged CA and CCPR. The latter include a combination of ischemic injury (i.e., CA), hypoxic injury (i.e., CCPR with low flow and low pressure), and reperfusion/reoxygenation injury [18,33,34,35]. A correlation between survival and the duration of CCPR and CA has been described [7,15], and consistent, intact neurological survival is unlikely after prolonged CA (>10–15 min) and/or prolonged CCPR (>20–30 min) [7,15]. Our data showed age-dependent survival rates up to 44% in patients with CCPR for 30–60 min and up to 29% in the subgroup of CCPR > 60 min (Supplementary Materials, Table S6).

To improve the results after CCPR, extracorporeal circulation for cardiopulmonary resuscitation (ECPR) has been used for 15 years in order to restore perfusion and oxygenation, and many studies have been published that evaluate this approach. However, in the past, the results have often been inconsistent and the criteria for inclusion/exclusion vary widely between studies, leading to generally low evidence levels and the potential for bias [36,37,38].

Registry studies with ECPR for OHCA have reported survival rates of 27.6% [39] and survival with a good neurologic outcome between 9% and 33% [40,41,42]. During the last two years, three randomized controlled studies have been published for OHCA that compare ECPR with CCPR [43,44,45]. The ARREST trial [45] showed a significantly better intact neurological survival for an ECPR group than for a CCPR group (43% vs. 7%), whereas PRAGUE OHCA showed no overall survival benefit with a neurologically favorable outcome at 180 days (31.5% vs. 22%), even though there was a significantly improved neurological survival after 30 days (30.6 vs. 18.2%). In the specific setting of the INCEPTION trial, no significant difference between ECPR (20%) and CCPR (16%) with respect to intact neurological survival was described [44].

Our study characteristics differed from those of the three published randomized controlled trials [43,44,45] and the previous registry studies. It is important to note that our study had fewer exclusion criteria and included all rhythms, unwitnessed OHCA, and patients older than 65–70 years (Supplementary Materials, Table S4). Despite these differences in patient profiles, the overall survival in the subgroup of our OHCA patients (n = 40) of 35% (Supplementary Materials, Table S5) was better than that of the INCEPTION trial [44], similar to that of the PRAGUE OHCA study, and not significantly different from that of the ARREST trial cohort (43%) [45]. Our 3- and 6-month survival with good neurological outcomes (CPC 1 and 2) (27.5%) was better than that of the INCEPTION trial (17%) [44], comparable with that of the Prague OHCA study (31.5%) [43], and worse than the ARREST trial (43%).

For IHCA, registry studies and metadata analyses comparing ECPR and CCPR showed a significantly better overall and intact neurological survival for ECPR, ranging between 20% and 30.4% [16,36,46,47]. Our own IHCA data suggested an even further improvement, with an overall survival rate at hospital discharge of 52% and an intact neurological survival (CPC 1–2) at 3 months of 41% (Table 2). The cause for this improved outcome for IHCA vs. OHCA patients was most likely due to the shorter CCPR duration for these patients (Table 3).

Our data for patients with OHCA who were cannulated pre-hospital pointed in the same direction. Survival at hospital discharge of the 14 patients with pre-hospital cannulation was 57%, which was favorable compared with a previously published survival of 8.4% [5] and a survival with CPC 1–2 of 28.6% [48].

As a historical control group from our own institution, we used retrospective registry data published in 2017 that provided details of the results after ECPR (n = 133) over a period of five years [49]. The IHCA survival (n = 74) was 18.9% and the OHCA survival (n = 59) was 8.5%; the duration of CCPR was strongly correlated with survival [49]. Our current survival data (IHCA survival 52% and OHCA survival 23%; Table 2) showed an improvement in survival in favor of our multi-organ repair approach.

Our data supported the fact that the duration of CCPR is a critical risk factor for survival after CA. Therefore transportable, mobile devices might be advantageous for an earlier treatment initiation. Our data indicated that femoral cannulation could rapidly be performed (56% in <15 min) (Table 3). Pre-hospital cannulation was associated with a short CCPR duration (mean of 31 min), very good survival (57%), and acceptable complication rates at experienced centers (Supplementary Materials, Table S1), especially those already familiar with ECPR. Nevertheless, pre-hospital cannulation requires special technical and staff resources [5]. In contrast, patients after OHCA who were cannulated in-hospital had a mean CCPR duration of 75 min and survival rate of 23.1% (Table 2 and Table 3). A comparison of the effects of CCPR duration and age on outcome (Supplementary Table S6) showed the best results in patients between 18 and 64 years with a CCPR duration < 30 min, a finding supported by many other studies. However, our data also showed satisfactory survival in patients aged 65–75 years in all three CCPR duration groups (<30 min: 100%; 30–60 min: 40%; >60 min: 29%). Due to the small number of patients (n = 4) older than 75 years and CCPR < 30 min, no meaningful conclusion could be drawn, even though survival was excellent (100%).

The clinical events documented in patients undergoing the CARL treatment (Supplementary Materials, Table S1) such as bleeding, ischemia, and neurological events were consistent with those reported in other studies [50,51]. The most common complication (approximately 30%, with a range between 10 and 60%) after ECPR is bleeding [50,52,53], which depends on various factors such as previous co-morbidities, the anticoagulation regimen, and the types of cannulas used. This was also the most frequently reported event in our study, mostly related to coagulopathy (61%), the cannulation site (25%), and surgical procedures (28%) and often occurring in combination (Supplementary Materials, Table S1). The rate of pseudosubarachnoid hemorrhage (pSAH) was 10% (7/69) (Table S1) and this did not have any therapeutic consequences. The incidence of pSAH is generally higher in patients with pre-existing cerebrovascular disease or with longer periods of low blood flow to the brain [52,54,55]. However, the incidences of renal failure (13%), infections (12%), generalized inflammatory reactions (7%), and hemolysis (3%) were observed less frequently than in previous reports (acute kidney injury, 63% [56]; infections, 8–23% [50,51]). Vascular complications with leg ischemia have been reported to occur in up to 17% [5,47,57]; the incidence in our series was <9% (Table S1). The absence of thrombotic complications in our study might have been related to the presence of heparin in the cytoprotective solution, the administration of dual antiplatelet therapy (DAPT) in the catheterization laboratory in patients undergoing percutaneous coronary interventions, and the generally low level of coagulation after CA.

### Limitations of the Study

Our study was subject to certain limitations inherent to observational research. These limitations included a small sample size, which is an issue common to many other studies on CA and CCPR. Furthermore, as this is the first clinical report on a novel treatment strategy for refractory CA, we were not able to employ randomization. Instead, we opted for an all-comers prospective study design, including all consecutive patients who met the minimal inclusion characteristics. As we had no control group, we discussed our data from previous CCPR results and historical ECPR results from our own institution and the findings from published registries and three randomized trials.

We are aware that there was a potential selection bias in this study, which is inherent in any registry study. Beyond that, in the field of research focusing on this special population of CPR/ECPR patients, data are heterogenous in terms of outcomes and reporting. Even randomized trials are subject to bias due to (a) very variable baseline inclusion criteria for potentially treatable patients, (b) heterogeneous local and country-specific emergency medical systems, and (c) the impossibility of blinding and frequent crossovers in randomized studies.

There are no registries available with reliable data on the incidence of IHCA and OHCA CPR/ECPR at the different participating centers. Beyond that, the COVID-19 pandemic caused the reallocation of hospital resources, which severely hampered timely patient recruitment at the centers. It was also reported that survival of OHCA was worse during the COVID-19 pandemic [58].

Adherence to the complete study protocol varied among the centers, with each highly experienced center relying on its established routines that had already been in place for many years. We anticipate that stricter adherence to the protocol and increased experience with the new technique could further enhance patient outcomes. Given the limited number of patients, we did not explore potential center-specific differences.

Although this study did not enforce strict inclusion and exclusion criteria, future trials and registries will help to establish parameters for improved application. Such research efforts are vital to enhance our understanding, improving the application of this innovative treatment strategy and thereby impacting daily treatment practices for a large population.

## 5. Conclusions

Our results showed that (1) controlled automated reperfusion of the whole body has the potential to further improve outcomes after prolonged refractory CCPR, (2) cardiac, renal, hepatic, and pulmonary functions may be restored, and (3) further studies are necessary to define the clinical relevance of this first report in various subgroups.

## Figures and Tables

**Table 1:** Baseline demographics and pre-hospital resuscitation characteristics (n = 69).

Characteristic	Parameter	Value
Age	[years], mean ± SD Range	59.0 ± 13.4 21–86
	18–64 years, n (%)	46 (67)
	65–75 years, n (%)	18 (26)
Sex	Male, n (%)	54 (78.3)
	Female, n (%)	15 (21.7)
Weight	[kg], median (IQR) (n = 56)	84 (80–96)
Medical history	Previous illnesses known, n (%)	55 (79.7)
	Coronary artery disease, n (%)	26 (37.7)
	Cardiomyopathy, n (%)	8 (11.6)
	Cerebrovascular stenoses, n (%)	3 (4.3)
	Other cardiovascular diseases, n (%)	18 (26.1)
	Diabetes, n (%)	10 (14.5)
	Respiratory, n (%)	3 (4.3)
	Substance abuse, n (%)	14 (20.3)
	Renal insufficiency, n (%)	5 (7.2)
	Cancer, n (%)	4 (5.8)
	Rheumatic, n (%)	2 (2.9)
	Other, n (%)	31 (44.9)
Etiology of CA	Myocardial infarction, n (%)	43 (62.3)
	Aortic dissection type A, n (%)	7 (10.1)
	Pulmonary arterial embolism, n (%)	4 (5.8)
	Arrhythmogenic, n (%)	4 (5.8)
	Cardiomyopathy, n (%)	3 (4.3)
	Valvular heart disease (aortic stenosis), n (%)	1 (1.4)
	Hypoxia, n (%)	3 (4.3)
	Intoxication, n (%)	2 (2.9)
	Drowning, n (%)	1 (1.4)
	Hypovolemia, n (%)	1 (1.4)
Implanted AED	Internal defibrillator, n (%)	2 (2.9)
	External defibrillator (vest), n (%)	0 (0)
Location of CA	IHCA, n (%)	29 (42)
	OHCA, n (%)	40 (58)
	Medical facility, n (%)	2 (5.0)
	Home/residence, n (%)	13 (32.5)
	Street/highway, n (%)	9 (22.5)
	Industrial/workplace, n (%)	6 (15.0)
	Sports/recreation event, n (%)	5 (12.5)
	Public building, n (%)	3 (7.5)
	Other, n (%)	2 (5.0)
Unwitnessed CA	n (%)	8 (11.6)
Witnessed CA	Bystander witnessed, n (%)	29 (42.0)
	Of those, bystander CPR, n (%)	22 (75.9)
	EMS witnessed, n (%)	32 (46.4)
AED use among n = 22 patients	AED used	1 (4.5)
Initial rhythm monitored at CA in n = 62 patients	Ventricular fibrillation, n (%)	29 (46.8)
	Pulseless ventricular tachycardia, n (%)	4 (6.5)
	Asystole, n (%)	10 (16.1)
	Pulseless electrical activity, n (%)	19 (30.6)
Airway management in n = 64 patients	Endotracheal tube, n (%)	58 (90.6)
	Supraglottic airway, n (%)	3 (4.7)
	Surgical airway, n (%)	2 (3.1)
	Not used, n (%)	1 (1.6)
Mechanical CPR device used in n = 56 patients	n (%)	36 (64.3)
Defibrillations in n = 49 patients	[Number per patient], median (IQR)	3 (IQR 0–5)
End-tidal CO_2_ during CPR	[mmHg], median (IQR) (n = 11)	25 (IQR 20–30)
pH before CARL *	Mean ± SD (n = 21)	7.0 ± 0.3
Lactate before CARL *	[mmol/L], median (IQR) (n = 21)	10.1 (7.7–12.3)

* The CARL Controller contains the option for online venous and arterial blood gas analysis. Therefore, blood gas analysis was available in patients connected to the CARL system before the start of extracorporeal circulation. N = 69 unless otherwise noted. CA: cardiac arrest; AED: automated external defibrillator; OHCA: out-of-hospital cardiac arrest; IQR: interquartile range; IHCA: in-hospital cardiac arrest; EMS: emergency medical service; CPR: cardiopulmonary resuscitation; CARL: controlled automated reperfusion of the whole body; SD: standard deviation.

**Table 2:** Primary outcomes and duration of treatment, ICU stay, and hospital stay.

Characteristic	Parameter	Value
IHCA + OHCA	Overall survival at hospital discharge, n (%)	29 (42)
	Deemed “non-survivable”, n (%)	9 (13)
	Survival among remaining, n (%)	29 (48)
	Survival at 3 months with CPC 1–2, n (%)	23 (33)
IHCA (n = 29)	Overall survival at hospital discharge, n (%)	15 (52)
	Survival at 3 months with CPC 1–2, n (%)	12 (41)
OHCA (n = 40)	Overall survival at hospital discharge, n (%)	14 (35)
	Survival at 3 months with CPC 1–2, n (%)	11 (28)
	Survival at hospital discharge of patients	
	transported to the hospital (n = 26), n (%)	6 (23)
	Survival at hospital discharge of patients	
	cannulated pre-hospital (n = 14), n (%)	8 (57)
Primary cause of death among n = 40 deceased	Neurological, n (%)	10 (25.0)
	Multiple organ failure, n (%)	11 (27.5)
	Hemorrhage, n (%)	3 (7.5)
	Cardiac pump failure, n (%)	4 (10.0)
	Acute aortic dissection type A, n (%)	7 (17.5)
	Left ventricular rupture, n (%)	2 (5.0)
	Sepsis, n (%)	0 (0)
	Other, n (%)	3 (7.5)
ICU stay (n = 62)	[days], median (IQR)	8.1 (1.5–23.0)
Hospital stay	[days], median (IQR)	10 (1–28)
	Among deceased patients [days], median (IQR) (n = 40)	1 (0–6)
	Among survivors [days], median (IQR) (n = 29)	28 (16–49)

N = 69 unless otherwise noted. ICU: intensive care unit; CPC: cerebral performance category; OHCA: out-of-hospital cardiac arrest; IHCA: in-hospital cardiac arrest.

**Table 3:** Time intervals and locations before, during, and after CA and CPR (n = 69).

Time Interval	Number of Patients *	Value
Time from emergency call to EMS arrival (OHCA) [min], mean ± SD	26/40	8.9 ± 4.9
Time from call to first shock [min], median (IQR)	15/69	12.4 (6.5–19.5)
Duration of CCPR in all patients [min], median (IQR)		
IHCA + OHCA	62/69	51.5 (30.0–74.5)
IHCA	26/62	33.5 (18.5–49.3)
OHCA	36/62	68.5 (43.8–81.3)
Pre-hospital cannulation	11/36	31.0 (20.0–56.5)
In-hospital cannulation	25/36	75.0 (67.0–88.0)
Duration of CCPR only for OHCA, n (%)		
<30 min	36	5 (13.9)
30–60 min	36	9 (25.0)
>60 min	36	22 (61.1)
Time until cannulation was established (IHCA and OHCA), n (%)		
<10 min	41	4 (9.8)
10–15 min	41	19 (46.3)
16–20 min	41	6 (14.6)
21–25 min	41	6 (14.6)
>25 min	41	6 (14.6)
Time from CA to start of CARL (IHCA + OHCA) [min], mean ± SD	54/69	59.2 ± 30.8

* Number of patients with data/all patients in the relevant group. There was a varying availability of values, which was caused by multiple interfaces in the treatment process. Therefore, the number of patients with available values for the given data point is provided in relation to the given collective (for further details, see Supplementary Methods). CA: cardiac arrest; CCPR: conventional cardiopulmonary resuscitation; OHCA: out-of-hospital cardiac arrest; IHCA: in-hospital cardiac arrest; CARL: controlled automated reperfusion of the whole body.

**Table 4:** Assessment of organ functions at timepoints after cardiac arrest.

Organ System	24 h	7 Days	30 Days	Hospital Discharge
Number of patients assessed at timepoint	44	32	18	23
**Kidney**				
Creatinine [mg/dL], median (IQR)	1.44 (1.03–1.99)	1.30 (0.96–2.65)	0.98 (0.75–1.82)	0.75 (0.65–0.95)
Patients with data, n (total)	32	29	18	19
GFR [mL/min], mean ± SD	58.2 ± 22.5	63.7 ± 31.5	78.9 ± 52.0	82.7 ± 32.6
Patients with data, n (total)	20	15	7	9
Renal replacement therapy, n (%)	4 (9.8)	9 (28.1)	2 (11.1)	1 (4.3)
Patients with data, n (total)	41	32	18	23
**Liver**				
AST/GOT [U/L], median (IQR)	273 (165–384)	101 (70–312)	43 (34–70)	25 (21–37)
Patients with data, n (total)	31	20	9	9
ALT/GPT [U/L], median (IQR)	103 (49–195)	69 (60–144)	5 (53–102)	31 (22–86)
Patients with data, n (total)	32	18	11	11
**Respiratory System**				
Invasive ventilation, n (%) *	44 (100)	20 (63)	6 (33)	3 (13)
Patients with data, n (total)	44	32	18	23
Non-invasive ventilation, n (%)	0 (0)	2 (6)	1 (6)	0 (0)
Patients with data, n (total)	44	32	18	23
**Cardiovascular System**				
LV functional impairment				
None, n (%)	4 (9.1)	11 (34.4)	4 (22.2)	8 (34.8)
Mild, n (%) **	1 (2.3)	1 (3.1)	0 (0)	3 (13.0)
Moderate, n (%) **	7 (15.9)	1 (3.1)	0 (0)	1 (4.3)
Severe, n (%) **	19 (43.2)	7 (21.9)	4 (22.2)	3 (13.0)
No values available, n (%)	13 (29.5)	12 (37.5)	10 (5.6)	8 (34.8)
Patients with data, n (total)	44	32	18	23
Dependent on inotropes	34 (79.1)	9 (28.1)	2 (11.1)	1 (4.5)
Patients with data, n (total)	43	32	18	
Dependent on vasopressors	39 (88.6)	12 (37.5)	2 (11.1)	0 (0)
Patients with data, n (total)	44	32	18	23

* CARL was used beyond acute treatment as veno-arterial extracorporeal circulation for continued short-term support. As an increasing number of centers favor awake patients, weaning from the respirator followed by non-invasive ventilator support was sometimes faster than weaning from the extracorporeal circulation. ** Assessment of cardiac function was semi-quantitative and according to the recommendation of the American Heart Association: no functional impairment = LVEF 50–70%; mild impairment = LVEF 41–49%; moderate impairment = LVEF 30–40%; severe impairment = LVEF < 30%. ALT: alanine aminotransferase; AST: aspartate aminotransferase; CARL: controlled automated reperfusion of the whole body; GFR: glomerular filtration rate; GOT: glutamic oxaloacetic transaminase; GPT: glutamic pyruvic transaminase; IQR: interquartile range; LV: left ventricular; SD: standard deviation.

## Data Availability

All data can be accessed upon reasonable request from the following repository: mandated accession code: https://freidok.uni-freiburg.de/data/237340 (accessed on 14 December 2023).

## Supplementary Materials

The following supporting information can be downloaded at: https://www.mdpi.com/article/10.3390/jcm13010056/s1 [59,60,61,62,63,64,65,66,67,68,69,70,71,72,73,74,75,76,77,78,79,80,81,82,83,84,85,86,87,88,89,90,91,92,93,94,95,96,97,98,99,100,101,102,103,104,105,106,107,108,109,110,111,112,113,114,115,116,117,118,119,120,121,122,123,124,125,126,127,128,129,130,131,132,133,134,135,136,137,138]. Table S1: Summary of clinical events documented in patients treated with multi-organ repair after CA. Table S2: Components of the cytoprotective solution (priming solution) and additives. Table S3: STROBE Statement—checklist (separate file). Table S4: Study characteristics for ARREST, Prague OHCA, INCEPTION, and CARL. Table S5: Outcomes after OHCA in ARREST vs. Prague OHCA vs. INCEPTION compared with CARL. Table S6: Survival in relation to age and duration of CCPR. Table S7: Consideration of differences between CCPR, ECPR, and CARL. Figure S1: Pericyte control of cerebral blood flow. Figure S2: Panels A–D: Flowchart of the comprehensive study protocol. Box S1: Rationale for multi-organ repair. Box S2: Comprehensive study protocol details. Box S3: Detailed description of 2 patients with ventricular rupture and 9 patients with type A dissection.
