# Supplementary material for: Treatment of Refractory Cardiac Arrest by Controlled Reperfusion of the Whole Body: A Multicenter, Prospective Observational Study
Source: J Clin Med. 2023 Dec 21;13(1):56. doi: 10.3390/jcm13010056 (PMC10780178; doi:10.3390/jcm13010056)
Supplement: Supplementary file 1 [file jcm-13-00056-s001.zip › jcm-2738017-supplementary.pdf]

Supplementary Information to

"Treatment of Refractory Cardiac Arrest by Controlled Reperfusion of the Whole Body: A Multicenter, Prospective Observational Study"

Contents

Supplementary Methods

Supplementary Tables S1-S7

Supplementary Table S1: Summary of clinical events documented in patients treated with multi-organ repair after CA

Supplementary Table S2: Components of the cytoprotective solution (priming solution) and additives

Supplementary Table S3: STROBE Statement - checklist (separate file)

Supplementary Table S4: Study characteristics for ARREST, Prague OHCA, INCEPTION, and CARL

Supplementary Table S5: Outcomes after OHCA in ARREST vs. Prague OHCA vs. INCEPTION compared with CARL

Supplementary Table S6: Survival in relation to age and duration of CCPR

Supplementary Table S7: Consideration of differences between CCPR, ECPR, and CARL

Supplementary Figures S1-S2

Supplementary Figure S1: Pericyte control of cerebral blood flow

Supplementary Figure S2 Panels A-D: Flowchart of the comprehensive study protocol

Supplementary Boxes S1-S3

Supplementary Box S1: Rationale for multi-organ repair

Supplementary Box S2: Comprehensive study protocol details

Supplementary Box S3: Detailed description of 2 patients with ventricular rupture and 9 patients with type A dissection

Supplementary Information References

SUPPLEMENTARY METHODS

Clinical design, data, and site monitoring

The sponsor (Resuscitec GmbH, Freiburg, Germany) had no role in the study design, site selection, data collection and analysis, monitoring or writing of the manuscript. Written informed consent by the patients or their legal representatives was mandatory for all data collection and processing (for further details see below under "Patients"). The fundamental considerations for the study size were the primary and secondary endpoints within the framework of a combined phase I/II clinical trial.

Data and site monitoring, data collection and data analysis were performed by an independent consulting and research company in the field of health care technology (Novineon CRO & Consulting Ltd., Tübingen, Germany). The authors designed the comprehensive study protocol (**Supplementary Information, Box 2** and **Figure S2**).

We adhered to the guidelines set forth by the Strengthening the Reporting of Observational Studies in Epidemiology (STROBE) statement to ensure the comprehensive reporting of our findings [1].

As part of this study, we included the STROBE Statement checklist of items (**Supplementary Information, Table S3).**

The denominator for several parameters was not 69 patients, i.e., not 100%. This is because there are multiple interfaces between out-of-hospital emergency medical services, hospitals, emergency rooms, catheter laboratories, and intensive care units with no continuous documentation at any of the participating centers. In combination with the highly life-threatening disease of acute CA followed by CCPR investigated here, the quality of corresponding data documentation varied between centers and individual patients. Furthermore, strict monitoring by the CRO allowed the entry of only conceivable documented values and no acceptance of estimated values.

Patients

To prevent selection bias, we designed an all-comers study without formalized patient selection, i.e., regardless of age, duration of CCPR, or transport time, and even patients with unwitnessed CA and "un-survivable" diseases were included. Basic and advanced life support techniques were delivered as rapidly as possible. If there was no sustained ROSC after 10-15 min, the OHCA patients were either transported to the

next suitable hospital or the EMS team was alerted and drove to the patient in a rendezvous system. The decision to continue with CCPR or to use whole-body reperfusion was made by the EMS team at the location of CA. CARL treatment was started (a) if there was still refractory CA and no stable ROSC, (b) based on given guidelines, expert consensus statements, and generally accepted indications and contraindications for ECPR [2, 3], and (c) at the discretion of the responsible physician [4, 5]. Contraindications were stable ROSC upon arrival of the CARL team.

Since this was a non-interventional study, rather than inclusion or exclusion criteria currently accepted indications and contraindications for the use of extracorporeal circulation in cases involving CCPR following CA were applied. Therefore, the following contraindications are not specific for extracorporeal multi-organ treatment or the registry study itself but are derived from the current state-of-the-art in ECPR [2, 3].

Contraindications were limited to factors that represent general contraindications for any invasive treatment or prevent the implementation of the multi-organ therapy:

- “Do not resuscitate” request of the patient
- Stable ROSC upon arrival of the CARL team
- Inability to cannulate the femoral vessels
- Septic shock
- Known severe chronic neurological impairment
- Patients with a known diagnosis of end-stage cancer
- Inability to achieve the minimal standards of extracorporeal perfusion (low flow, low pressure) and/or multi-organ protection

List and definitions of primary and secondary endpoints

The primary endpoints were defined as overall survival at hospital discharge and good neurological outcome (CPC score 1 or 2) at hospital discharge. The CPC scale ranges from 1 (good cerebral performance) to 5 (brain death). A favorable CPC score is defined as ≤ 2. The clinical effectiveness endpoints were the number of patients who survived and the number of patients who survived with good neurological outcome.

The following secondary endpoints were assessed:

- Bleeding, except cerebral: defined if any intervention (e.g., transfusion, surgical repair, etc.) following this event (bleeding) was necessary. The differentiation between bleeding events from the cannula side and coagulopathy was performed by the subjective evaluation at the participating centers.
- Cerebral bleeding: this endpoint required the proof of a CT scan. Any cerebral bleeding was noted and included, regardless of size and location of the bleeding.
- Successful arterial and venous cannulation: defined as anatomically correct cannulation allowing connection to the CARL Controller and establishment of sufficient blood flow.
- Controlled oxygenation (100 – 200 mmHg): attaining the defined arterial oxygen levels by using the online blood gas analyzer and adequate adaptation of the oxygen supply.
- Pulsatile flow: the automated generation of a pulsatile flow was documented in the CARL Controller data sheet.
- Target $PaCO_2$ of 40 – 45 mmHg: this was achieved by adaptation of the sweep-gas flow.
- Target pH in first 30 min < 7.25; thereafter 7.35 – 7.45: this was achieved by monitoring with the blood gas analyzer and adaptation of the arterial pH.
- Target mean arterial blood pressure (60 – 100 mmHg): this was achieved by using the invasive, fiberoptic blood pressure monitoring catheter and appropriate medication.
- Mild systemic hypothermia (32-33°C): this state was achieved by the hypothermic device and monitored in real time by a cuvette in the venous bloodline.
- Kidney function: measured by the glomerular filtration rate (GFR) and the necessity for renal replacement therapy, as documented in the patient’s chart.
- Acute kidney injury: presumed a creatinine increase of 1.5-fold baseline or increase of > 26.3 µmol / 0.3 mg/dL within 48 hours.
- Liver function: measured by the enzyme activity of AST/ALT, as documented in the patient’s chart.
- Pulmonary function: measured by blood gases and the need for mechanical ventilation, as documented in the patient’s chart.

- Cardiac and hemodynamic functions: these were semi-quantitatively assessed by documentation of inotropic agent use in patient’s chart. Assessment of inotrope use at days 1, and 7, or of either CARL or ECLS termination, at day 30 and at hospital discharge.
- Presence of infections: this was registered if proof via microbiological culture or laboratory values was obtained.
- Duration (in hours) of controlled automated reperfusion of the whole body.
- Duration of ICU stay in hours as documented in the patient’s chart.
- Duration of hospital stay (in days): Time in hospital between admission (for OHCA) or occurrence of CA (for IHCA) and release from any hospital treatment as documented in the patient’s chart.

Statistical analysis

Data and site monitoring, data collection and analysis were performed by an independent consulting and research company in the field of health care technology (Novineon CRO & Consulting Ltd., Tübingen, Germany). The adjudication of primary and secondary endpoints was performed by an independent committee of physicians. For the primary endpoint, follow-up was 100% complete.

For data analysis the following software packages were used: R versions 4.2.3 (2023-03-15) -- "Shortstop Beagle" and 4.1.2 (2021-11-01) – “Bird Hippie” were used for analysis (Copyright (C) 2023 The R Foundation for Statistical Computing, Platform: x86_64-w64-mingw32/x64 (64-bit); RStudio 2023.03.0 Build 386 "Cherry Blossom" Release (3c53477a, 2023-03-09) for Windows, Mozilla/5.0 (Windows NT 10.0; Win64; x64) AppleWebKit/537.36 (KHTML, like Gecko) RStudio/2023.03.0+386 Chrome/108.0.5359.179 Electron/22.0.3 Safari/537.3; RStudio 2022.07.0+548 "Spotted Wakerobin" Release (34ea3031089fa4e38738a9256d6fa6d70629c822, 2022-07-06) for Windows, Mozilla/5.0 (Windows NT 10.0; Win64; x64) AppleWebKit/537.36 (KHTML, like Gecko) QtWebEngine/5.12.8 Chrome/69.0.3497.128 Safari/537.36).

The datasets generated and analyzed during the current study are available in the https://freidok.uni-freiburg.de/data/237340 repository. These datasets are available

from the corresponding author on reasonable request. All data generated or analyzed during this study on patients treated for acute cardiac arrest are included in this published article and its supplementary information files.

Mandated accession code

https://freidok.uni-freiburg.de/data/237340

SUPPLEMENTARY TABLES S1 – S7

Supplementary Table S1: Summary of clinical events documented in patients treated with extracorporeal multi-organ treatment after CA * (n=69)

Event type	Event description	Number of events
Bleeding**	Related to coagulopathy	42/69
	Related to veno-arterial cannulation	17/69
	Related to ICU/surgical procedures	19/69
	Other	3/69
Neurologic	Cerebral ischemia	10/69
	Hypoxic-ischemic encephalopathy	9/69
	Brain edema	1/69
	Pseudosubarachnoid hemorrhage***	7/69
Ischemia	Peripheral, related to femoral cannulation	6/69
	Abdominal	6/69
	Thrombosis/Vascular spasm	4/69
Renal	Acute kidney injury****	9/69
Infection*****	All sites	8/69
Generalized inflammatory reaction		5/69
Hemolysis		2/69
Other		3/69

** Potential side effects during treatment using extracorporeal multi-organ technology and the necessary femoral cannulation were registered as "events" in all N=69 patients. To maximize surveillance of potential side effects within the study, multiple mentions of single events were possible, therefore indicating up to N=5 events in individual patients. Displayed numbers are related to a total of N=151 events.*

***Bleeding was defined if any intervention (e.g., transfusion, surgical repair, etc.) following this event (bleeding) had to be carried out.*

****Pseudosubarachnoid hemorrhage (pSAH) was minor and detected in routine CT scan (contrast enhancement) without any therapeutic consequences.*

*****Acute kidney injury presumed a creatinine increase of 1.5-fold of baseline or an increase of > 26.3 µmol / 0.3 mg/dL within 48 hours.*

******Infections were registered if documented by microbiological culture or laboratory values.*

Supplementary Table S2: Components of the cytoprotective solution (priming solution) and additives

Substance	Trade name (manufacturer)	Concentration/ volume
Mannitol	CARL Priming Solution<br>Köhler Chemie, Bensheim, Germany	96.0 g/l
Sodium citrate dihydrate	CARL Priming Solution<br>Köhler Chemie, Bensheim, Germany	15.3 g/l
Magnesium chloride hexahydrate	CARL Priming Solution<br>Köhler Chemie, Bensheim, Germany	8.5 g/l
Heparin	Heparin sodium<br>B.Braun Melsungen, Germany	5,000 IU/1 ml
Lidocaine hydrochloride	Xylocaine<br>Aspen Pharma, Dublin, Ireland	500.0 mg/25 ml
Human albumin	Albunorm<br>Octapharma, Langenfeld, Germany	45 g/600 ml
Ascorbic acid	Pascorbin<br>Pacoe, Gießen, Germany	7.5 g/50 ml

Supplementary Table S3: STROBE Statement - Checklist

See separate file

Supplementary Table S4. Study characteristics for ARREST, Prague OHCA, INCEPTION, and CARL

	ARREST *	Prague OHCA*	INCEPTION*	CARL
Setting	Single-centre, University of Minnesota, USA	Single-centre, Charles University, Prague, Czech Republic	Multi-centre; the Netherlands	Multi-centre; Europe
Studied intervention	ECPR and early revascularization	Invasive bundle consisting of intra-arrest transport with mechanical CPR, ECPR, and immediate invasive assessment (coronary angiography)	ECPR	CARL
Comparator	CCPR and early revascularization	CCPR with encouraged immediate invasive assessment	CCPR	—
Number of participating centres	1	1	10	7
Inclusion period	August 2019 to June 2020	March 2013 to October 2020	May 2017 to February 2021	March 2020 to December 2022
Inclusion criteria				
Age	18-75 years	18-65 years	10-70 years	>18 years
Only witnessed OHCA	No	Yes	Yes	No
Rhythm	VR or pulseless VT	Presumed cardiac aetiology, all rhythms	VT/VF or AED-shock admitted	All rhythms
ROSC	No ROSC after 3 shocks	No ROSC after 5 min of ALS	No ROSC within 15 min of ALS	No ROSC within 15 min of ALS
Other	Body habitus able to support mechanical CPR, estimated transport time <30 min	ECPR team available at cardiac centre	Only bystander ALS	—
Exclusion criteria				
Mechanisms	Drowning, blunt or penetrating trauma, burns, overdose	Presumed non-cardiac cause	Non-shockable, multi-trauma	DNR, septic, hemorrhagic CA
Co-morbidities	Terminal cancer	Obvious life-threatening co-morbidities, known CPC >3, suspected/confirmed stroke	Oncological disease, bilateral femoral vessel surgery, CPC score >3, NYHA III/IV, COPD GIII/IV	End-stage cancer
Bleeding	Active GI or internal bleeding	Bleeding diathesis	—	Massive hemorrhagic CA
Other	Prisoner or nursing home resident; cath lab unavailable	Suspected or confirmed pregnancy, conscious patient	Expected initial cannulation >60 min after arrest, ROSC with	—

			sustained haemodynamic recovery within 15 min	
ECMO cannulation location	Catheterization lab	Catheterization lab	Emergency department	Catheterization lab, Emergency department or outside the hospital
Randomization				
Timing	Upon hospital arrival	On scene in collaboration with trial coordinator	During transport by hospital staff	No randomization
Blinding	EMS blinded	None	EMS blinded	None
Crossover	Allowed both directions	Allowed both directions	Allowed both directions	n.a.
Consent	As soon as feasible following randomization (patient or family)	Legal representative or patient after regaining neurological function	Deferred consent	Legal representative or patient
Primary outcome	Survival to hospital discharge	180-day survival with favourable neurological status (CPC1-2)	30-day survival with favourable neurological status (CPC1-2)	Neurological favourable survival (CPC1-2); hospital discharge and after 90 days

*Data taken from Ubben et al. [6]

Abbreviations: AED, automated external defibrillator; ALS, advanced life support; CPC, cerebral performance category; CCPR, conventional cardiopulmonary resuscitation; ECMO, extracorporeal membrane oxygenation; ECPR, extracorporeal cardiopulmonary resuscitation; EMS, emergency medical service; GI, gastrointestinal; NYHA, New York Heart Association; OHCA, out-of-hospital cardiac arrest; ROSC, return of spontaneous contractions; VF, ventricular fibrillation; VT, ventricular tachycardia; n.a., not applicable.

Supplementary Table S5. Outcomes after OHCA in ARREST vs. Prague OHCA vs. INCEPTION compared with CARL.

Outcomes	CCPR/standard treatment	ECPR/invasive strategy	*P*-value
**ARREST***	**n=15**	**n=14**	
Survival to hospital discharge	1 (7%)	6 (43%)	0.023
3-month survival	0	6 (43%)	0.006
6-month survival	0	6 (43%)	0.006
**Prague OHCA***	**n=132**	**n=124**	
30-day survival (CPC1-2)	24 (18.2%)	38 (30.6%)	0.02
6-month survival (CPC1-2)	29 (22.0%)	39 (31.5%)	0.09
**INCEPTION***	**n=64**	**n=70**	
30-day survival (CPC1-2)	10 (16%)	14 (20%)	0.518**
3-month survival (CPC1-2)	9 (14%)	12 (17%)	0.600**
6-month survival (CPC1-2)	10 (16%)	14 (20%)	0.537**
**CARL**	**n.a.**	**n=40**	**n.a.**
Survival at hospital discharge		14 (35%)	
CPC1-2 at discharge		8 (20%)	
3-month survival (CPC1-2)		11 (27.5%)	
6-month survival (CPC1-2)		11 (27.5%)	

*Data taken from Ubben et al. [6]
**62 patients with available data in CCPR group (30-day survival); 63 in CCPR group (3- and 6-month survival); 68 in ECPR group (3-month survival). [6]
Abbreviations: CPC, cerebral performance category; CCPR, conventional cardiopulmonary resuscitation; ECPR, extracorporeal cardiopulmonary resuscitation; OHCA, out-of-hospital cardiac arrest, n.a., not applicable.

Supplementary Table S6: Survival in relation to age and duration of CCPR

Age	CCPR < 30 min	CCPR 30 - 60 min	CCPR > 60 min
**18 – 64**<br><br>N=46/69	N= 6<br><br>Survival 100% (6/6)	N=16<br><br>Survival 44% (7/16)	N=19<br><br>Survival 11% (2/19)
**65 – 75**<br><br>N=18/69	N=4<br><br>Survival 50% (2/4)	N=5<br><br>Survival 40% (2/5)	N=7<br><br>Survival 29% (2/7)
**> 75**<br><br>N=5/69	N=4<br><br>Survival 100% (4/4)	N=1<br><br>Survival 0% (0/1)	N=0

Abbreviation: CCPR, conventional cardiopulmonary resuscitation

Supplementary Table S7: Consideration of differences between CCPR, ECPR, and CARL

Parameter	CCPR	ECPR	CARL
ECC			
° veno-arterial perfusion and oxygenation	/	X	X
° high flow		(X)	X
° pulsatility	/	/	X
° high pressure	/	(X)	X
° controlled oxygenation	/	(X)	X
Individualized multi-organ repair			
Control of the composition of the reperfusate			
° reduced calcium	/	/	X
° normal sodium	/	/	X
° increased potassium	/	/	X
° increased magnesium	/	/	X
° lower viscosity	/	/	X
° hemodilution	/	/	X
° increased osmolarity	/	/	X
° increased oncotic pressure	/	/	X
° lowering oxygen	/	/	X
° permission elevated CO_2	/	/	X
° pH-stat	/	/	X
° lidocaine	/	/	X
° heparin (anticoagulation)	/	X	X
° free radical scavengers	/	/	X
Control of the conditions of reperfusion			
° high flow		(X)	X
° pulsatility	/	/	X
° high pressure	/	(X)	X
° mobile, immediate hypothermia (resource-independent)	/	/	X
Online monitoring			
Hemodynamics			
° cardiac output	/	/	X
° heart rate	/	/	X
° blood pressure	/	/	X
Venous and arterial blood gas			
° pO_2	/	/	X
° pH	/	/	X
Electrolytes			
° calcium	/	/	X
° sodium	/	/	X
° potassium	/	/	X
Temperature			
° venous blood temperature	/	/	X

Legend: / = absent; (X) = sometimes present; X = present

SUPPLEMENTARY FIGURES S1-S2

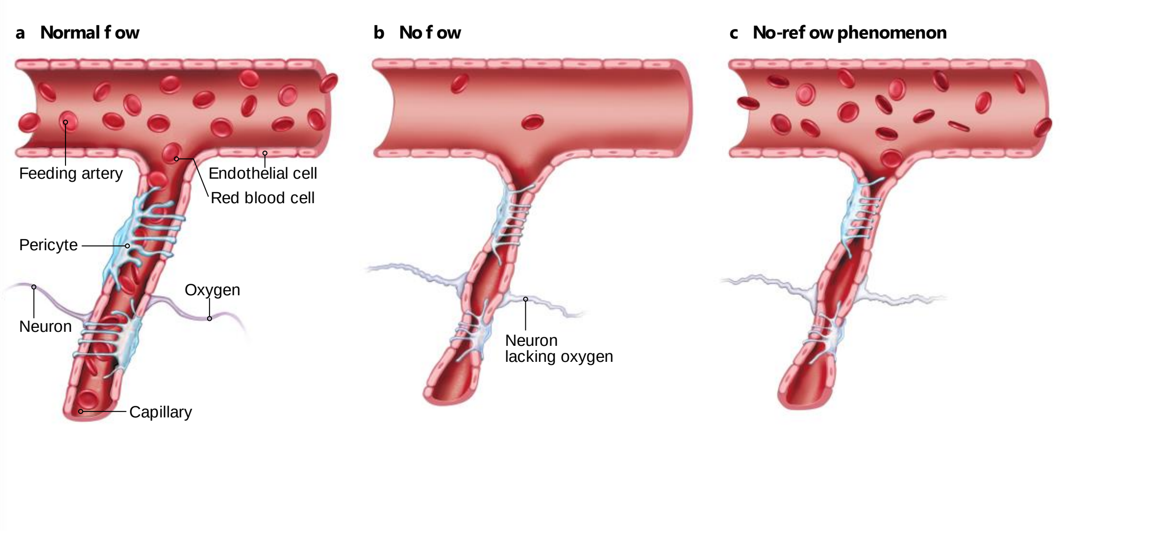

Supplementary Figure S1: Pericytes control cerebral blood flow (modified from Greif and Eichmann [7]; used with permission)

Panel A:

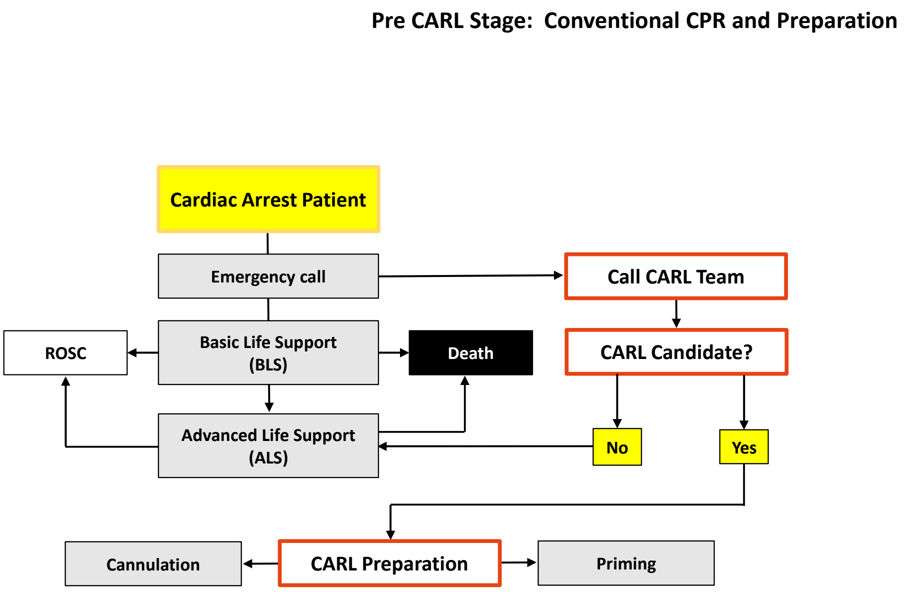

Panel B:

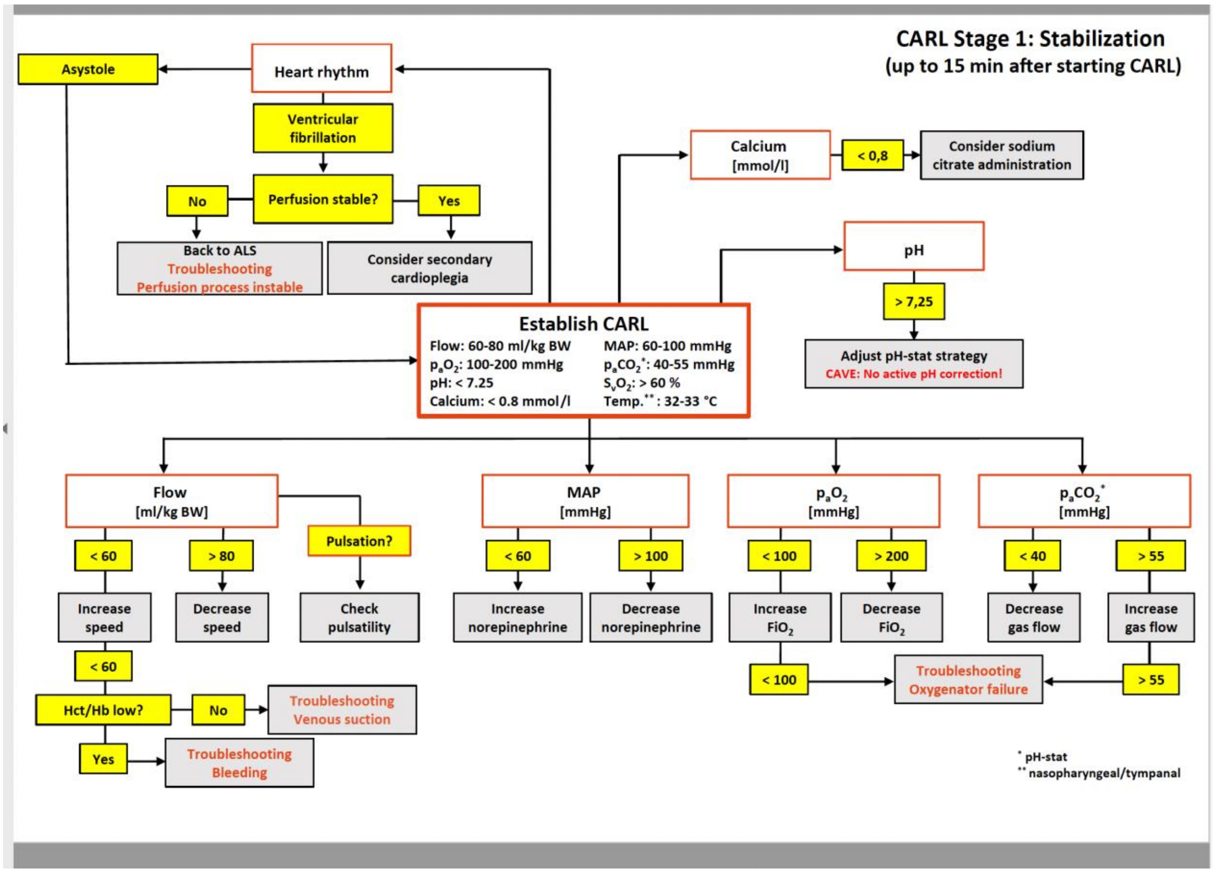

Panel C:

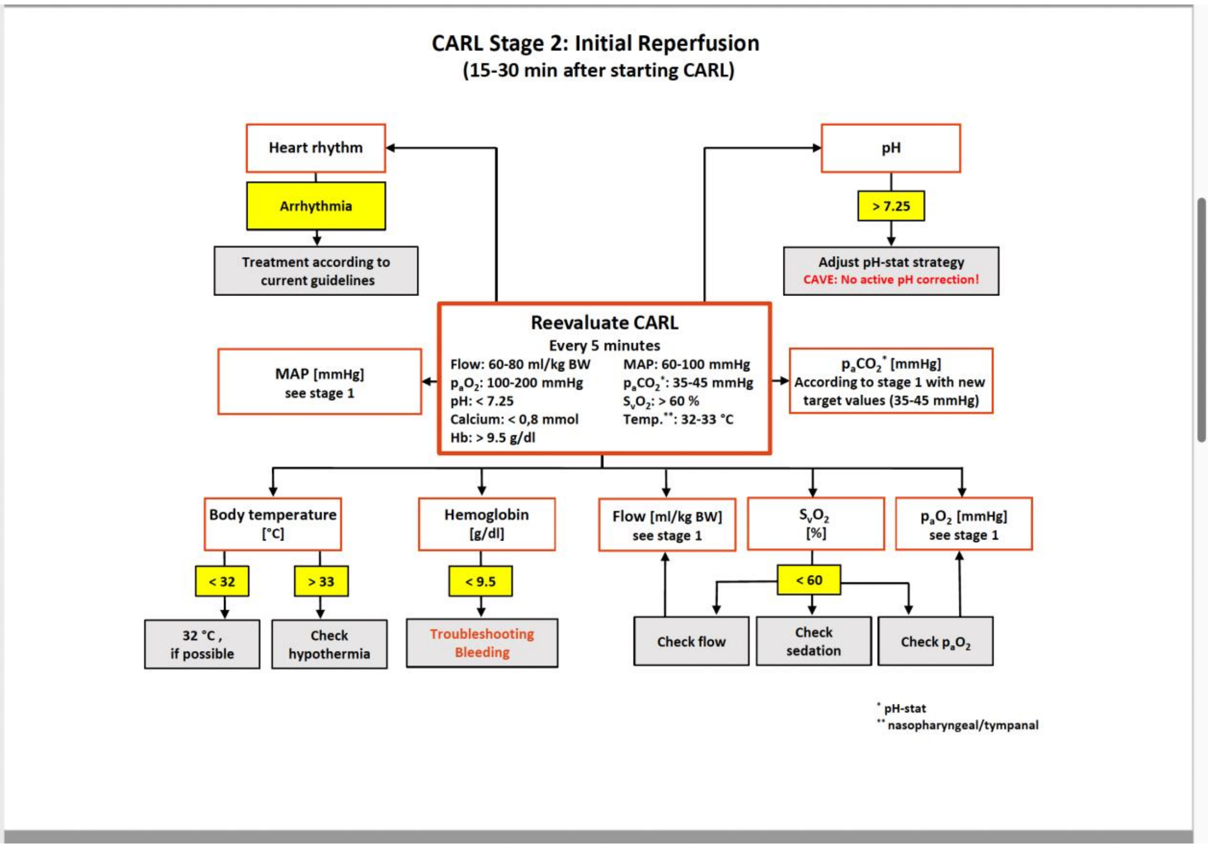

Panel D:

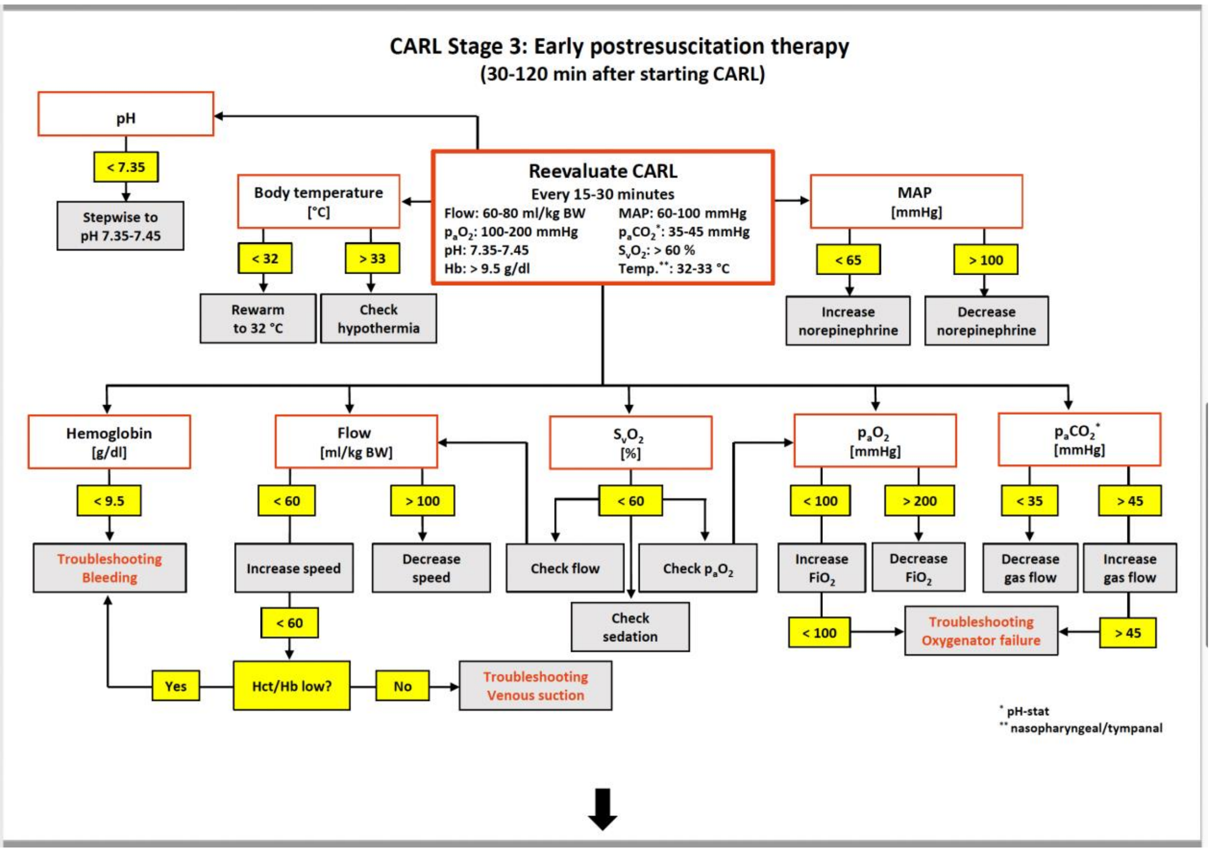

Supplementary Figure S2 A-D. Flowchart of the comprehensive study protocol

Supplementary Box S1. Rationale for multi-organ repair

Conditions of the reperfusion

Extracorporeal circulation is employed to counteract cardio-circulatory failure by providing immediate high pressure and pulsatile flow along with controlled oxygenation.

° Pulsatility

Pulsatility is achieved by using a 2-pump system to increase perfusion and reduce no-reflow phenomenon [8, 9].

° High blood pressure and high blood flow

The optimal pressure for the initial reperfusion varies between organs: whereas the heart and lungs require a low pressure, the brain needs higher pressure [10, 11]. It is therefore of utmost importance to find an optimal pressure for the entire body. Since the brain is the most vulnerable organ after cardiac arrest (CA), we have used 80 – 100 mmHg as the target pressure.

In a recent randomized study in patients who had been resuscitated from CA from Copenhagen [12], a mean pressure of 63 mmHg (low) was compared to 77 mmHg (high) and no difference was found. This was explained by the fact that both pressures were rather in the low range.

Systolic reperfusion pressure of 90 mmHg results in better cerebral performance category (CPC) and improved preserved cerebral mitochondrial viability (citrate synthase activity and cerebral cortical mitochondrial protein expression for fusion) [13].

High mean arterial pressure (80-100 mmHg) (as well as high osmolarity, anticoagulation and buffer) is needed to prevent the no-reflow phenomenon [14] and increase cerebral blood flow (CBF). Studies have shown that pericytes regulate cerebral blood flow and that the lack of oxygen causes pericyte cell death and capillary constriction [7, 15] (**Supplementary Information, Figure S1**). Pericyte relaxation allows for the dilatation of cerebral capillaries in response to neuronal activity (and in response to glutamate), whereas ischemia and hypoxia result in pericyte necrosis and capillary strangulation even after blood flow has been reestablished. Additionally, endothelial cell swelling further reduces capillary flow. Non-pulsatile extracorporeal perfusion results in hypotension and disturbances in cerebral autoregulation [16-18], leading to poor outcome after CA [19]. Our group has demonstrated that this can be prevented by generating high, pulsatile flow and resultant high blood pressure [8].

Hossmann [11] reported that a high cerebral blood flow above 40 ml/100g/min is needed for EEG recovery. However, it should be mentioned that an immediate rise in

high pressure might result in postischemic tissue vulnerability, which can be avoided by gradually raising the pressure after ischemia and avoiding pressure above 180 mmHg [11].

High arterial pressure is also needed to address specific anatomical properties of the highly sensitive hippocampus as the core part of the limbic system. The hippocampal areas are reported to have fewer perfused blood vessels than the cerebral cortex [20]. With respect to a cerebral low-flow or low-pressure situation, which may occur during CPR, this anatomical peculiarity may enhance a no-reflow phenomenon, aggravating hippocampal alterations [21].

High blood flow is necessary to obtain improved neurological recovery and overcome endothelial swelling especially in the cerebral circulation [11, 14]. However, this might lead (in patients with slight aortic insufficiency and even in those without) to left ventricular (LV) distention, endoventricular stasis and pulmonary edema, if the LV is not ejecting. Therefore, LV venting (preferably percutaneous) should be implemented to overcome this potential problem [22].

The CARL-specific invasive arterial blood pressure monitoring in the descending aorta allows a real-time assessment of myocardial function and therefore immediate adaptation of therapy towards unloading of the LV.

° Immediate mild hypothermia

Mild hypothermia (33-34°C) was achieved within 10 minutes after starting CARL therapy; this was achieved in approx. 70% of patients. Temperature has significant consequences for cellular processes of ATP production, and hypothermia reduces metabolic rate and may result in improved recovery after cerebral ischemia [11] and was therefore recommended in several previous studies [23-25] . Nevertheless, there is an ongoing debate about the induction of mild hypothermia after cardiopulmonary resuscitation.

The 2021 guidelines for the management of CA patients after ROSC recommended the use of targeted temperature management for unresponsive adults after ROSC [26]. A recent Cochrane Review also indicates that mild hypothermia potentially improves neurological outcome after cardiac arrest [27]. Contrary, another systematic review showed no benefit with respect to survival or functional outcome after OHCA [28]. In addition, a metaanalysis including 8 randomized controlled trials found better survival when compared to the subgroup of uncontrolled normothermia but showed no beneficial effects when compared to actively controlled normothermia [29].

No data are currently available concerning the importance of timing of induction of mild hypothermia, but experimental studies have shown outcome improvements if hypothermia is induced already during CCPR [30] and delay in cooling negates the beneficial effects of hypothermia in dogs [31] and in a randomized clinical trial (time to randomization 135 min, reaching targeted temperature (~33°C) after 7.5 hrs) [32].

Composition of the reperfusate

Multi-organ repair to reverse ischemic/hypoxic and reperfusion/reoxygenation injury is based on managing 14 components of the initial reperfusate composition and 4 parameters during reperfusion (**Supplementary Information Box S1**). The composition of the returning femoral venous blood is regulated by a unique cytoprotective solution (**Supplementary Information, Table S2**), and medication delivery is facilitated through a specialized delivery port (**Figure 1**). Controlled oxygenation (100-200 mmHg) is essential to prevent free radical formation [33-35]. To enable individualized real-time adjustment of physiological parameters, comprehensive real-time monitoring, and medication delivery are used.

° Ca^{++}

Ischemia leads to cytosolic calcium flooding and mitochondrial calcium overload, which induces apoptosis and programmed cell death [11]. Reducing ionized calcium in the initial reperfusate will help minimize intracellular accumulation of deleterious calcium and counteract calcium-mediated apoptosis.

° Na^{+}

Physiological sodium is intended to avoid excessive Na^{+} changes to prevent cerebral edema.

° K^{+}

Recent data from the DOSE VF Trial [36] have shown that the defibrillation strategy has a significant impact on the survival to hospital discharge and the neurological outcome. In a cluster-randomized trial, patients with refractory ventricular fibrillation (VF) during out-of-hospital cardiac arrest (OHCA) were treated with one of three different defibrillation strategies: double sequential external defibrillation (DSED; rapid sequential shocks from two defibrillators), vector-change defibrillation (VCD; switching the defibrillation pads to an anterior-posterior position) or standard defibrillation. Survival to hospital discharge was higher in the DSED and VCD groups than in the standard group (30.4%, 21.7% and 13.3%, resp.). DSED, but not VCD, was associated with a higher likelihood of a good neurological outcome than standard defibrillation [37].

Instead of multiple defibrillations we used systemic hyperkalemia (8 – 10 MEq/l) in some patients. The use of hyperkalemia to treat intractable VF stems from established cardiac surgical techniques (warm terminal reperfusion, secondary cardioplegia). This maneuver caused pharmacological asystole (arrest) with subsequent spontaneous resumption of heart action. Our previous experimental studies have shown that the success rate of defibrillation after intractable VF after CA and acute myocardial infarction is significantly higher after potassium delivery as compared with electrical defibrillation [38, 39] and this strategy has also been applied clinically [40, 41]. Systemic potassium administration was only used when ventricular fibrillation was the primary rhythm, and it seems to be a valuable option also for intractable VF after CA.

° Magnesium

Hypermagnesemia helps in membrane stabilization.

° Viscosity

Hyperviscosity improves perfusion and helps to prevent the no-reflow-phenomenon.

° Hemodilution

Hemodilution improves perfusion and helps to prevent the no-reflow-phenomenon.

° Increased osmotic pressure

During ischemia, brain tissue osmolality rises by about 50 mosm [14]. Favorable results have been obtained using albumin in the initial reperfusate [42] which improves perfusion and helps to prevent the no-reflow-phenomenon.

° Increased oncotic pressure

Increased oncotic pressure limits cerebral edema and decreases vasopressor requirements.

° Lowered pO_2

Detrimental effects of higher FiO_2 (> 0.6 – 0.8%) during general anesthesia [43] have been described to lead to increased levels of redox biomarkers which have been associated with adverse clinical outcome in a variety of clinical settings, including cardiac disease [44] and post-resuscitation situations [45]. Some studies suggest that the oxidative stress due to higher FiO_2 may occur within the pulmonary vasculature [46]. It is well known that hyperoxia induces increased reactive oxygen species (ROS) which in turn leads to acute lung injury and increased permeability of the alveolar/vascular interface and endothelial disruptions (also mediated by interleukins, cytokines, and chemokines) [47]. Early severe hyperoxia (> 300 mmHg) on ECPR is reported to be a significant risk factor for acute brain injury and mortality [34].

Timing also seems to be an issue. Hyperoxia used shortly after CA has been associated with an increased risk of ischemic encephalopathy and death in observational studies and animal studies [48-51]. However, no difference was found between a restrictive and a liberal oxygen target of partial pressure of arterial oxygen (PaO_2) when used in comatose adult OHCA patients who had been admitted to the hospital after being resuscitated after OHCA and who had a sustained return of spontaneous circulation (ROSC) [51]. The potential pathophysiological link between brain injury and oxygenation seems to occur in the early period after CA and to be driven by reperfusion injury with mitochondrial dysfunction and tissue inflammation [51-53]. Experimental data have demonstrated that this injury may be exacerbated by hyperoxemia [49].

Therefore, partial oxygen pressure (pO_2) is set to reach values between 100 – 200 mmHg. Some experience is required to adjust the value using both ventilation and oxygenator.

° Elevated pCO_2

Increased CO_2 is required for a pH-stat strategy.

° pH

Arterial pH is of great importance for postischemic recovery [11]. We used a pH-stat regimen, i.e., constant pH level at the patient´s temperature to lower the cellular metabolic rate.

° Lidocaine

Lidocaine (500 mg) is used to support systemic hyperkalemia (or electrical countershocks) to regain a stable cardiac rhythm, because prolonged VF will ultimately result in irreversible cardiac damage. It is a sodium channel blocker that reduces intracellular sodium and calcium accumulation. The broad therapeutic margin of lidocaine in combination with potential neuroprotective effects [54] supports this approach for CARL therapy.

° Anticoagulation by heparin

Anticoagulation is needed to counteract any thrombus formation after CA, CCPR, and extracorporeal circulation.

° Free radical scavengers

In order to minimize damage from oxygen-derived free radicals (ROS) and reactive nitrogen species (RNS), free radical scavengers have important physiological roles (e.g., as a transmitter, the innate immune system uses ROS to attack invading pathogens. Moderate increases in ROS lead to a greater production of ROS scavengers (hormesis), but higher ROS concentrations can also lead to tissue damage and organ failure (“oxidative stress”) [43, 55], which occurs regularly after CA and CPR [56]. The addition of free radical scavengers to the cytoprotective CARL solution should help to reduce the oxidative stress and to minimize damage from oxygen-derived free radicals [33, 35, 55].

While extracorporeal circulation, individualized multi-organ repair, and monitoring are ongoing, a safe time window opens immediately for diagnosing and treating of the underlying (mostly cardiac) cause of CA, either in the catheterization laboratory or in the operating room.

Supplementary Box S2. Comprehensive study protocol

This study protocol (see also flowchart in **Supplementary Figure S2 Panels A-D**) describes the rationale for application of extracorporeal multi-organ repair after refractory CA.

Our strategy includes (1) extracorporeal cardiopulmonary support, (2) personalized multi-organ repair, (3) comprehensive monitoring, (4) fast diagnosis and treatment of the underlying disease while multi-organ repair is ongoing, (5) the option for out-of-hospital treatment, and (6) translational research for clinical application.

Introduction

The high incidence of acute cardiac arrest (CA), accompanied by high mortality and neurological sequelae in the few survivors, represents an ongoing challenge in healthcare [5]. Regardless of the cause of CA, conventional cardiopulmonary resuscitation (CCPR) has been the treatment of choice for decades to counteract the life-threatening loss of circulation and respiratory function. However, during CCPR there are major challenges that compromise success, including a) that the underlying cause of CA cannot be treated on site, b) the absence of a reestablished spontaneous circulation despite maximized efforts of emergency medical services and therefore c) an ongoing period of a global ischemia followed by inadequate perfusion generated by chest compressions. As a consequence of this dilemma and despite extensive effort in research and education of CPR in recent years, the outcome of affected patients in terms of survival and neurological recovery has been unsatisfactory over decades [5, 57, 58].

To improve the results after CA, “targeted CPR” (tCPR) was introduced in which specific hemodynamic, respiratory, and metabolic targets have been defined that should be reached during the resuscitative efforts [59-62]. Among others, compression depth of cardiac massage, arterial blood pressure, end-tidal CO_2 measurement and the titration of oxygen are core elements of tCPR [62, 63]. Although tCPR represents a logical step towards a rationale-based and controlled therapeutic approach to CPR, due to a significant lack of available appropriate monitoring and related therapeutic interventions, the implementation of tCPR remains rudimentary.

Similar limitations also apply to the increasing use of extracorporeal circulatory life support systems (VA-ECMO, ECLS) during CCPR (extracorporeal CPR - ECPR). Although blood circulation and respiratory function may be replaced quickly by ECLS, the lack in comprehensive monitoring and personalized treatment of the ischemic/hypoxic and reperfusion/reoxygenation injury also applies to ECPR [64]. Consequently, survival and neurologic recovery of patients treated with ECPR has only changed to a limited extent [65-67]. Nevertheless, the entity of ECPR is a step toward improving the prognosis of resuscitated patients and has therefore been integrated in specific CCPR algorithms or as “alternative to conventional CPR in selected patients“ in current applicable guidelines [5, 68]. This framework of an insufficient therapeutic approach to a major healthcare problem promotes research and development of new ideas for providing better survival and neurologic recovery of affected patients. For this reason and based on the best available knowledge in this complex field, the

subsequently described concept of “Controlled reperfusion of the whole body” (CARL) has been developed over the past 15 years by our group.

The Concept of Controlled Automated Reperfusion of the Whole Body (CARL)

Ischemia/reperfusion and hypoxic/reoxygenation injury has been a topic of a continued research for decades. The core principle behind this injury is the fact, that deprivation of blood flow (ischemia and hypoxia) causes a time-dependent cellular damage mainly related to a lack of required substrates [69]. Reestablishing blood flow (reperfusion) to a former ischemic/hypoxic area provides primarily a new supply of nutrients for cellular metabolism; however, the extent of cellular damage further increases during the phase of reperfusion. The effect of this continued cellular damage is related to a disparity in actual demand and supply of the damaged cells during reperfusion. Moreover, reperfusion injury is triggered within brief periods of seconds or minutes following the reinstitution of blood flow to the ischemic tissue, therefore adding the requirement of a timely intervention of counteractive measures. The comprehension of these mechanisms and the derived therapeutic options limiting this injury has become an established part of therapeutic regimen in cardiac surgery, solid organ transplantation and limb reperfusion [70-73].

Core elements within therapeutic approaches to limit tissue damage are a) gaining control over the physical conditions of reperfusion (i.e. blood pressure, blood flow, pulsatility, body temperature) and b) the composition of the reperfusion solution, namely recirculating blood (i.e. O_2 and CO_2 levels, pH, electrolyte content, and osmolality) [8, 39, 74-80]. As mentioned above, readily available, and ample monitoring plays a key role in any therapeutic approach in these acute and critically sick patients. Therefore, the extent and demand for a suitable monitoring were defined in parallel to the conditions of reperfusion and the composition of the recirculating blood. Because of these demands, online and immediate blood gas analysis and arterial blood pressure monitoring have been implemented in our treatment modality, providing the treating team baseline elementary information for a rationale-based method rather than an uncontrolled symptomatic therapy of CA.

Since CA is frequently associated primarily with myocardial pump failure, an extracorporeal circulation was chosen to take over basic circulatory and respiratory function within the therapeutic approach of whole-body reperfusion. The treatment of different organs potentially requires different reperfusion conditions and solutions and therefore may lead to contrary requirements. Exemplary for conflicting priorities in a setting of whole-body reperfusion is the element “arterial blood pressure” in myocardial and cerebral reperfusion, with the goal of achieving “gentle” blood pressures during myocardial reperfusion and comparable high blood pressure demand in cerebral reperfusion [10, 75] (see also **Supplementary Box S1**). Based on this background and acknowledging such conflicting demands, our concept was drafted based primarily on established elements for multi-organ repair after injury. Moreover, and apart from the focus of current CCPR practices, the severe impact of a generalized reperfusion injury of the whole body and the specific demand of the most sensitive organ, the brain, was of utmost importance in developing the concept of CARL.

The global concept of CARL and its individual elements were tested and further developed in numerous chronic animal experiments during the last 15 years [8, 39, 76-81]. A unique chronic animal model was established in order to investigate beneficial

components in the setting of global reperfusion after severe ischemia with respect to the endpoints mortality and neurological recovery [79]. The animals were exposed to periods of 15 minutes and 20 minutes of unprotected warm ischemia without any resuscitative attempts during this time [76, 78, 79]. The ensuring reperfusion period of 60 minutes was characterized by adapting the reperfusion conditions and the circulating blood according to the continuous and readily available monitoring (i.e., blood gas monitoring, arterial blood pressure, temperature measurement, mixed venous oxygen saturation etc.). Depending on the variable tested, up to 90% of the animals survived the experimental course with up to 90% of the survivors indicating a complete neurological recovery [76-80].

In view of the available publications concerning single-organ ischemia and the results of the described animal experiments incorporating the global approach of CARL, a set of targets to be met during the initial stage of reperfusion have been identified as beneficial. Continuous blood gas analysis is the starting point for a reasonable adaption of parameters like paO_2, $paCO_2$, pH, sodium, potassium, and calcium (see **Supplementary Box S1**). In addition, the presence of a continuous arterial blood pressure and flow monitoring allows continual adaption according to the individual needs of the patient, including the selection of approved infusion solutions.

Further detailed information is shown in Supplementary Figure S2: Panels A-D.

CARL Post-ROSC Treatment

The CARL post-ROSC treatment is based on a general concept comprising several routine elements. Blood pressure monitoring and online arterial and venous blood gas analysis were derived from the CARL system, providing sufficient monitoring. Systemic cooling was implemented immediately at the beginning of whole-body reperfusion, reaching the target temperature of 33 - 34°C within 10 minutes. pH stat with an arterial pH not above 7.25 was provided for 30 minutes followed by return to physiological values thereafter. Serum calcium content was lowered to 0.5 - 0.8 mmol/l using a special priming solution that contained further potentially protective solutions like magnesium, mannitol, lidocaine, ascorbic acid, and human albumin. High blood pressure and pulsatile flow (5-6 l/min) were continued as long asystole/PEA or ventricular fibrillation were present. With the occurrence of ROSC, blood flow and pressure delivered by the extracorporeal circulation was reduced to allow ejection from the left ventricle. Special care was taken to maintain normal oxygen levels (100-180 mmHg), avoiding detrimental hyperoxygenation during this phase. The online blood gas monitoring and a specialized gas blender are used for this purpose. Generally, normal physiological values are slowly reached after 60 minutes of reperfusion, when stable ROSC has been achieved. However, mild hypothermia of 33 - 34°C is maintained for 24 hours followed by stepwise rewarming to 36°C after 48 hours for neurological assessment.

CARL post-ROSC treatment is incorporated in the setting of critical care following CPR and fulfills guideline requirements with respect to counteracting post-resuscitation syndrome and all other aspects of critical care [82]. Overall, best-practice recommendations for post-ROSC treatment should be provided, including neurological assessment and limiting individual and center-specific protocols.

Please also refer to “**Supplementary Information Fig. S2 Panel D**” and “**Supplementary Box S1**”.

Supplementary Box S3. Detailed description of 2 patients with ventricular rupture and 7 patients with type A dissection

---

Some out-of-hospital EMS teams were equipped with portable ultrasound devices and were therefore able to detect relevant findings (i.e., pericardial or pleural effusion, visible signs of intraaortic dissection), and some staff was trained by a Focused Echocardiography in Emergency Life Support (FEEL) course. Since the trauma room in this study was considered as the final part of EMS treatment at this relevant interface between out-of- and in-hospital critical care, these suspected individual diagnoses were confirmed immediately thereafter by CT scan.

Ventricular rupture
Two patients experienced a CA due to myocardial rupture caused by a preceding myocardial infarction. Rapid femoral cannulation (N=1 IHCA, N=1 OHCA) resulted in rapid diagnosis. However, due to pericardial tamponade combined with the acute massive exsanguination, sufficient circulation could not be achieved. Despite extensive surgical repair of the corresponding defects, the postoperative course was characterized by severe and irreversible multi-organ failure, ultimately resulting in the patients' death.

Acute type A dissection and subsequent CA
In 4/7 patients, a diagnosis of acute type A dissection was confirmed by a CT scan immediately following the implantation of CARL system. In the remaining 3/7 patients, clinical signs like acute and very severe chest and back pain coupled with sonographic proof of pericardial tamponade were clear indicators of an acute aortic dissection. In the given situations, the treating teams of these seven patients decided to discontinue the therapy due to its perceived futility.

Supplementary Material References

1. von Elm, E.; Altman, D. G.; Egger, M.; Pocock, S. J.; Gøtzsche, P. C.; Vandenbroucke, J. P., The Strengthening the Reporting of Observational Studies in Epidemiology (STROBE) statement: guidelines for reporting observational studies. *Ann Intern Med* **2007,** 147, (8), 573-7.

2. Kim, H.; Cho, Y. H., Role of extracorporeal cardiopulmonary resuscitation in adults. *Acute Crit Care* **2020,** 35, (1), 1-9.

3. Soar, J.; Maconochie, I.; Wyckoff, M. H.; Olasveengen, T. M.; Singletary, E. M.; Greif, R.; Aickin, R.; Bhanji, F.; Donnino, M. W.; Mancini, M. E.; Wyllie, J. P.; Zideman, D.; Andersen, L. W.; Atkins, D. L.; Aziz, K.; Bendall, J.; Berg, K. M.; Berry, D. C.; Bigham, B. L.; Bingham, R.; Couto, T. B.; Böttiger, B. W.; Borra, V.; Bray, J. E.; Breckwoldt, J.; Brooks, S. C.; Buick, J.; Callaway, C. W.; Carlson, J. N.; Cassan, P.; Castrén, M.; Chang, W. T.; Charlton, N. P.; Cheng, A.; Chung, S. P.; Considine, J.; Couper, K.; Dainty, K. N.; Dawson, J. A.; de Almeida, M. F.; de Caen, A. R.; Deakin, C. D.; Drennan, I. R.; Duff, J. P.; Epstein, J. L.; Escalante, R.; Gazmuri, R. J.; Gilfoyle, E.; Granfeldt, A.; Guerguerian, A. M.; Guinsburg, R.; Hatanaka, T.; Holmberg, M. J.; Hood, N.; Hosono, S.; Hsieh, M. J.; Isayama, T.; Iwami, T.; Jensen, J. L.; Kapadia, V.; Kim, H. S.; Kleinman, M. E.; Kudenchuk, P. J.; Lang, E.; Lavonas, E.; Liley, H.; Lim, S. H.; Lockey, A.; Lofgren, B.; Ma, M. H.; Markenson, D.; Meaney, P. A.; Meyran, D.; Mildenhall, L.; Monsieurs, K. G.; Montgomery, W.; Morley, P. T.; Morrison, L. J.; Nadkarni, V. M.; Nation, K.; Neumar, R. W.; Ng, K. C.; Nicholson, T.; Nikolaou, N.; Nishiyama, C.; Nuthall, G.; Ohshimo, S.; Okamoto, D.; O'Neil, B.; Yong-Kwang Ong, G.; Paiva, E. F.; Parr, M.; Pellegrino, J. L.; Perkins, G. D.; Perlman, J.; Rabi, Y.; Reis, A.; Reynolds, J. C.; Ristagno, G.; Roehr, C. C.; Sakamoto, T.; Sandroni, C.; Schexnayder, S. M.; Scholefield, B. R.; Shimizu, N.; Skrifvars, M. B.; Smyth, M. A.; Stanton, D.; Swain, J.; Szyld, E.; Tijssen, J.; Travers, A.; Trevisanuto, D.; Vaillancourt, C.; Van de Voorde, P.; Velaphi, S.; Wang, T. L.; Weiner, G.; Welsford, M.; Woodin, J. A.; Yeung, J.; Nolan, J. P.; Hazinski, M. F., 2019 International Consensus on Cardiopulmonary Resuscitation and Emergency Cardiovascular Care Science With Treatment Recommendations: Summary From the Basic Life Support; Advanced Life Support; Pediatric Life Support; Neonatal Life Support; Education, Implementation, and Teams; and First Aid Task Forces. *Circulation* **2019,** 140, (24), e826-e880.

4. Richardson, A. S. C.; Tonna, J. E.; Nanjayya, V.; Nixon, P.; Abrams, D. C.; Raman, L.; Bernard, S.; Finney, S. J.; Grunau, B.; Youngquist, S. T.; McKellar, S. H.; Shinar, Z.; Bartos, J. A.; Becker, L. B.; Yannopoulos, D.; Bˇelohlávek, J.; Lamhaut, L.; Pellegrino, V., Extracorporeal Cardiopulmonary Resuscitation in Adults. Interim Guideline Consensus Statement From the Extracorporeal Life Support Organization. *Asaio j* **2021,** 67, (3), 221-228.

5. Soar, J.; Böttiger, B. W.; Carli, P.; Couper, K.; Deakin, C. D.; Djärv, T.; Lott, C.; Olasveengen, T.; Paal, P.; Pellis, T.; Perkins, G. D.; Sandroni, C.; Nolan, J. P., European Resuscitation Council Guidelines 2021: Adult advanced life support. *Resuscitation* **2021,** 161, 115-151.

6. Ubben, J. F. H.; Heuts, S.; Delnoij, T. S. R.; Suverein, M. M.; van de Koolwijk, A. F.; van der Horst, I. C. C.; Maessen, J. G.; Bartos, J.; Kavalkova, P.; Rob, D.; Yannopoulos, D.; Bělohlávek, J.; Lorusso, R.; van de Poll, M. C. G., Extracorporeal cardiopulmonary resuscitation for refractory OHCA: lessons from three randomized controlled trials-the trialists' view. *Eur Heart J Acute Cardiovasc Care* **2023,** 12, (8), 540-547.

7. Greif, D. M.; Eichmann, A., Vascular biology: Brain vessels squeezed to death. *Nature* **2014,** 508, (7494), 50-1.

8. Kreibich, M.; Trummer, G.; Beyersdorf, F.; Scherer, C.; Förster, K.; Taunyane, I.; Benk, C., Improved Outcome in an Animal Model of Prolonged Cardiac Arrest Through Pulsatile High Pressure Controlled Automated Reperfusion of the Whole Body. *Artif Organs* **2018,** 42, (10), 992-1000.

9. Shariff, M.; Dobariya, A.; Albaghdadi, O.; Awkal, J.; Moussa, H.; Reyes, G.; Syed, M.; Hart, R.; Longfellow, C.; Douglass, D.; El Ahmadieh, T. Y.; Good, L. B.; Jakkamsetti, V.; Kathote, G.; Angulo, G.; Ma, Q.; Brown, R.; Dunbar, M.; Shelton, J. M.; Evers, B. M.; Patnaik, S.; Hoffmann, U.; Hackmann, A. E.; Mickey, B.; Peltz, M.; Jessen, M. E.; Pascual, J. M., Maintenance of pig brain function under extracorporeal pulsatile circulatory control (EPCC). *Sci Rep* **2023,** 13, (1), 13942.

10. Okamoto, F.; Allen, B. S.; Buckberg, G. D.; Bugyi, H.; Leaf, J., Reperfusion conditions: importance of ensuring gentle versus sudden reperfusion during relief of coronary occlusion. *J Thorac Cardiovasc Surg* **1986,** 92, (3 Pt 2), 613-20.

11. Hossmann, K. A., Resuscitation potentials after prolonged global cerebral ischemia in cats. *Crit Care Med* **1988,** 16, (10), 964-71.

12. Kjaergaard, J.; Møller, J. E.; Schmidt, H.; Grand, J.; Mølstrøm, S.; Borregaard, B.; Venø, S.; Sarkisian, L.; Mamaev, D.; Jensen, L. O.; Nyholm, B.; Høfsten, D. E.; Josiassen, J.; Thomsen, J. H.; Thune, J. J.; Obling, L. E. R.; Lindholm, M. G.; Frydland, M.; Meyer, M. A. S.; Winther-Jensen, M.; Beske, R. P.; Frikke-Schmidt, R.; Wiberg, S.; Boesgaard, S.; Madsen, S. A.; Jørgensen, V. L.; Hassager, C., Blood-Pressure Targets in Comatose Survivors of Cardiac Arrest. *N Engl J Med* **2022,** 387, (16), 1456-1466.

13. Senthil, K.; Morgan, R. W.; Hefti, M. M.; Karlsson, M.; Lautz, A. J.; Mavroudis, C. D.; Ko, T.; Nadkarni, V. M.; Ehinger, J.; Berg, R. A.; Sutton, R. M.; McGowan, F. X.; Kilbaugh, T. J., Haemodynamic-directed cardiopulmonary resuscitation promotes mitochondrial fusion and preservation of mitochondrial mass after successful resuscitation in a pediatric porcine model. *Resusc Plus* **2021,** 6, 100124.

14. Hossmann, K. A.; Schmidt-Kastner, R.; Grosse Ophoff, B., Recovery of integrative central nervous function after one hour global cerebro-circulatory arrest in normothermic cat. *J Neurol Sci* **1987,** 77, (2-3), 305-20.

15. Hall, C. N.; Reynell, C.; Gesslein, B.; Hamilton, N. B.; Mishra, A.; Sutherland, B. A.; O'Farrell, F. M.; Buchan, A. M.; Lauritzen, M.; Attwell, D., Capillary pericytes regulate cerebral blood flow in health and disease. *Nature* **2014,** 508, (7494), 55-60.

16. Andersen, K.; Waaben, J.; Husum, B.; Voldby, B.; Bødker, A.; Hansen, A. J.; Gjedde, A., Nonpulsatile cardiopulmonary bypass disrupts the flow-metabolism couple in the brain. *J Thorac Cardiovasc Surg* **1985,** 90, (4), 570-9.

17. Borgdorff, P.; Sipkema, P.; Westerhof, N., Pump perfusion abolishes autoregulation possibly via prostaglandin release. *Am J Physiol* **1988,** 255, (2 Pt 2), H280-7.

18. Mendelowitz, D.; Scher, A. M., Pulsatile pressure can prevent rapid baroreflex resetting. *Am J Physiol* **1990,** 258, (1 Pt 2), H92-100.

19. Iijima, T.; Bauer, R.; Hossmann, K. A., Brain resuscitation by extracorporeal circulation after prolonged cardiac arrest in cats. *Intensive Care Med* **1993,** 19, (2), 82-8.

20. Kumar, K.; Goodrich, J.; Marcoux, F., Comparison of vascular perfusion in ischemia-sensitive and ischemia-resistant regions of gerbil brain by an automated laser cytometric device: a preliminary study. *J Neurosci Methods* **1991,** 39, (1), 1-8.

21. Shiroyama, Y.; Nagamitsu, T.; Yamashita, K.; Yamashita, T.; Abiko, S.; Ito, H., Changes in brain stem blood flow under various grades of brain stem ischemia. *Tohoku J Exp Med* **1991,** 164, (3), 237-46.

22. Pappalardo, F.; Montisci, A., What is extracorporeal cardiopulmonary resuscitation? *J Thorac Dis* **2017,** 9, (6), 1415-1419.

23. Mild therapeutic hypothermia to improve the neurologic outcome after cardiac arrest. *N Engl J Med* **2002,** 346, (8), 549-56.

24. Bernard, S. A.; Gray, T. W.; Buist, M. D.; Jones, B. M.; Silvester, W.; Gutteridge, G.; Smith, K., Treatment of comatose survivors of out-of-hospital cardiac arrest with induced hypothermia. *N Engl J Med* **2002,** 346, (8), 557-63.

25. Nolan, J. P.; Deakin, C. D.; Soar, J.; Böttiger, B. W.; Smith, G., European Resuscitation Council guidelines for resuscitation 2005. Section 4. Adult advanced life support. *Resuscitation* **2005,** 67 Suppl 1, S39-86.

26. Nolan, J. P.; Sandroni, C.; Böttiger, B. W.; Cariou, A.; Cronberg, T.; Friberg, H.; Genbrugge, C.; Haywood, K.; Lilja, G.; Moulaert, V. R. M.; Nikolaou, N.; Olasveengen, T. M.; Skrifvars, M. B.; Taccone, F.; Soar, J., European Resuscitation Council and European Society of Intensive Care Medicine guidelines 2021: post-resuscitation care. *Intensive Care Med* **2021,** 47, (4), 369-421.

27. Arrich, J.; Schütz, N.; Oppenauer, J.; Vendt, J.; Holzer, M.; Havel, C.; Herkner, H., Hypothermia for neuroprotection in adults after cardiac arrest. *Cochrane Database Syst Rev* **2023,** 5, (5), Cd004128.

28. Fernando, S. M.; Di Santo, P.; Sadeghirad, B.; Lascarrou, J. B.; Rochwerg, B.; Mathew, R.; Sekhon, M. S.; Munshi, L.; Fan, E.; Brodie, D.; Rowan, K. M.; Hough, C. L.; McLeod, S. L.; Vaillancourt, C.; Cheskes, S.; Ferguson, N. D.; Scales, D. C.; Sandroni, C.; Nolan, J. P.; Hibbert, B., Targeted temperature management following out-of-hospital cardiac arrest: a systematic review and network meta-analysis of temperature targets. *Intensive Care Med* **2021,** 47, (10), 1078-1088.

29. Sanfilippo, F.; La Via, L.; Lanzafame, B.; Dezio, V.; Busalacchi, D.; Messina, A.; Ristagno, G.; Pelosi, P.; Astuto, M., Targeted Temperature Management after Cardiac Arrest: A Systematic Review and Meta-Analysis with Trial Sequential Analysis. *J Clin Med* **2021,** 10, (17).

30. Sterz, F.; Safar, P.; Tisherman, S.; Radovsky, A.; Kuboyama, K.; Oku, K., Mild hypothermic cardiopulmonary resuscitation improves outcome after prolonged cardiac arrest in dogs. *Crit Care Med* **1991,** 19, (3), 379-89.

31. Kuboyama, K.; Safar, P.; Radovsky, A.; Tisherman, S. A.; Stezoski, S. W.; Alexander, H., Delay in cooling negates the beneficial effect of mild resuscitative cerebral hypothermia after cardiac arrest in dogs: a prospective, randomized study. *Crit Care Med* **1993,** 21, (9), 1348-58.

32. Dankiewicz, J.; Cronberg, T.; Lilja, G.; Jakobsen, J. C.; Levin, H.; Ullén, S.; Rylander, C.; Wise, M. P.; Oddo, M.; Cariou, A.; Bělohlávek, J.; Hovdenes, J.; Saxena, M.; Kirkegaard, H.; Young, P. J.; Pelosi, P.; Storm, C.; Taccone, F. S.; Joannidis, M.; Callaway, C.; Eastwood, G. M.; Morgan, M. P. G.; Nordberg, P.; Erlinge, D.; Nichol, A. D.; Chew, M. S.; Hollenberg, J.; Thomas, M.; Bewley, J.; Sweet, K.; Grejs, A. M.; Christensen, S.; Haenggi, M.; Levis, A.; Lundin, A.; Düring, J.; Schmidbauer, S.; Keeble, T. R.; Karamasis, G. V.; Schrag, C.; Faessler, E.; Smid, O.; Otáhal, M.; Maggiorini, M.; Wendel Garcia, P. D.; Jaubert, P.; Cole, J. M.; Solar, M.; Borgquist, O.; Leithner, C.; Abed-Maillard, S.; Navarra, L.; Annborn, M.; Undén, J.; Brunetti, I.; Awad, A.; McGuigan, P.; Bjørkholt Olsen, R.; Cassina, T.; Vignon, P.; Langeland, H.; Lange, T.; Friberg, H.; Nielsen, N., Hypothermia versus Normothermia after Out-of-Hospital Cardiac Arrest. *N Engl J Med* **2021,** 384, (24), 2283-2294.

33. Chouchani, E. T.; Methner, C.; Nadtochiy, S. M.; Logan, A.; Pell, V. R.; Ding, S.; James, A. M.; Cochemé, H. M.; Reinhold, J.; Lilley, K. S.; Partridge, L.; Fearnley, I. M.; Robinson, A. J.; Hartley, R. C.; Smith, R. A.; Krieg, T.; Brookes, P. S.; Murphy, M. P., Cardioprotection by S-nitrosation of a cysteine switch on mitochondrial complex I. *Nat Med* **2013,** 19, (6), 753-9.

34. Shou, B. L.; Ong, C. S.; Premraj, L.; Brown, P.; Tonna, J. E.; Dalton, H. J.; Kim, B. S.; Keller, S. P.; Whitman, G. J. R.; Cho, S. M., Arterial oxygen and carbon dioxide tension and acute brain injury in extracorporeal cardiopulmonary resuscitation patients: Analysis of the extracorporeal life support organization registry. *J Heart Lung Transplant* **2023,** 42, (4), 503-511.

35. Tomaselli, G. F.; Barth, A. S., Sudden cardio arrest: oxidative stress irritates the heart. *Nat Med* **2010,** 16, (6), 648-9.

36. Cheskes, S.; Verbeek, P. R.; Drennan, I. R.; McLeod, S. L.; Turner, L.; Pinto, R.; Feldman, M.; Davis, M.; Vaillancourt, C.; Morrison, L. J.; Dorian, P.; Scales, D. C., Defibrillation Strategies for Refractory Ventricular Fibrillation. *N Engl J Med* **2022,** 387, (21), 1947-1956.

37. Lim, G. B., Alternative defibrillation strategies improve outcomes. *Nat Rev Cardiol* **2023,** 20, (1), 5.

38. Beyersdorf, F.; Acar, C.; Buckberg, G. D.; Partington, M. T.; Okamoto, F.; Allen, B. S.; Bugyi, H. I.; Young, H. H., Studies on prolonged acute regional ischemia. IV. Aggressive surgical treatment for intractable ventricular fibrillation after acute myocardial infarction. *J Thorac Cardiovasc Surg* **1989,** 98, (4), 557-66.

39. Liakopoulos, O. J.; Allen, B. S.; Buckberg, G. D.; Hristov, N.; Tan, Z.; Villablanca, J. P.; Trummer, G., Resuscitation after prolonged cardiac arrest: role of cardiopulmonary bypass and systemic hyperkalemia. *Ann Thorac Surg* **2010,** 89, (6), 1972-9.

40. Athanasuleas, C. L.; Buckberg, G. D.; Allen, B. S.; Beyersdorf, F.; Kirsh, M. M., Sudden cardiac death: directing the scope of resuscitation towards the heart and brain. *Resuscitation* **2006,** 70, (1), 44-51.

41. Beyersdorf, F.; Kirsh, M.; Buckberg, G. D.; Allen, B. S., Warm glutamate/aspartate-enriched blood cardioplegic solution for perioperative sudden death. *J Thorac Cardiovasc Surg* **1992,** 104, (4), 1141-7.

42. Wengenmayer, T.; Schroth, F.; Biever, P. M.; Duerschmied, D.; Benk, C.; Trummer, G.; Kaier, K.; Bode, C.; Staudacher, D. L., Albumin fluid resuscitation in patients on venoarterial extracorporeal membrane oxygenation (VA-ECMO) therapy is associated with improved survival. *Intensive Care Med* **2018,** 44, (12), 2312-2314.

43. Oldman, A. H.; Martin, D. S.; Feelisch, M.; Grocott, M. P. W.; Cumpstey, A. F., Effects of perioperative oxygen concentration on oxidative stress in adult surgical patients: a systematic review. *Br J Anaesth* **2021,** 126, (3), 622-632.

44. Cabello, J. B.; Burls, A.; Emparanza, J. I.; Bayliss, S. E.; Quinn, T., Oxygen therapy for acute myocardial infarction. *Cochrane Database Syst Rev* **2016,** 12, (12), Cd007160.

45. Kilgannon, J. H.; Jones, A. E.; Shapiro, N. I.; Angelos, M. G.; Milcarek, B.; Hunter, K.; Parrillo, J. E.; Trzeciak, S., Association between arterial hyperoxia following resuscitation from cardiac arrest and in-hospital mortality. *Jama* **2010,** 303, (21), 2165-71.

46. Koksal, G. M.; Dikmen, Y.; Erbabacan, E.; Aydin, S.; Çakatay, U.; Sitar, M. E.; Altindas, F., Hyperoxic oxidative stress during abdominal surgery: a randomized trial. *J Anesth* **2016,** 30, (4), 610-9.

47. Bhandari, V., Molecular mechanisms of hyperoxia-induced acute lung injury. *Front Biosci* **2008,** 13, 6653-61.

48. Kilgannon, J. H.; Jones, A. E.; Parrillo, J. E.; Dellinger, R. P.; Milcarek, B.; Hunter, K.; Shapiro, N. I.; Trzeciak, S., Relationship between supranormal oxygen tension and outcome after resuscitation from cardiac arrest. *Circulation* **2011,** 123, (23), 2717-22.

49. Pilcher, J.; Weatherall, M.; Shirtcliffe, P.; Bellomo, R.; Young, P.; Beasley, R., The effect of hyperoxia following cardiac arrest - A systematic review and meta-analysis of animal trials. *Resuscitation* **2012,** 83, (4), 417-22.

50. Roberts, B. W.; Kilgannon, J. H.; Hunter, B. R.; Puskarich, M. A.; Pierce, L.; Donnino, M.; Leary, M.; Kline, J. A.; Jones, A. E.; Shapiro, N. I.; Abella, B. S.; Trzeciak, S., Association Between Early Hyperoxia Exposure After Resuscitation From Cardiac Arrest and Neurological Disability: Prospective Multicenter Protocol-Directed Cohort Study. *Circulation* **2018,** 137, (20), 2114-2124.

51. Schmidt, H.; Kjaergaard, J.; Hassager, C.; Mølstrøm, S.; Grand, J.; Borregaard, B.; Roelsgaard Obling, L. E.; Venø, S.; Sarkisian, L.; Mamaev, D.; Jensen, L. O.; Nyholm, B.; Høfsten, D. E.; Josiassen, J.; Thomsen, J. H.; Thune, J. J.; Lindholm, M. G.; Stengaard Meyer, M. A.; Winther-Jensen, M.; Sørensen, M.; Frydland, M.; Beske, R. P.; Frikke-Schmidt, R.; Wiberg, S.; Boesgaard, S.; Lind Jørgensen, V.; Møller, J. E., Oxygen Targets in Comatose Survivors of Cardiac Arrest. *N Engl J Med* **2022,** 387, (16), 1467-1476.

52. Bro-Jeppesen, J.; Johansson, P. I.; Kjaergaard, J.; Wanscher, M.; Ostrowski, S. R.; Bjerre, M.; Hassager, C., Level of systemic inflammation and endothelial injury is associated with cardiovascular dysfunction and vasopressor support in post-cardiac arrest patients. *Resuscitation* **2017,** 121, 179-186.

53. Wiberg, S.; Stride, N.; Bro-Jeppesen, J.; Holmberg, M. J.; Kjærgaard, J.; Larsen, S.; Donnino, M. W.; Hassager, C.; Dela, F., Mitochondrial dysfunction in adults after out-of-hospital cardiac arrest. *Eur Heart J Acute Cardiovasc Care* **2020,** 9, (4_suppl), S138-s144.

54. Leng, T.; Gao, X.; Dilger, J. P.; Lin, J., Neuroprotective effect of lidocaine: is there clinical potential? *Int J Physiol Pathophysiol Pharmacol* **2016,** 8, (1), 9-13.

55. Sies, H.; Jones, D. P., Reactive oxygen species (ROS) as pleiotropic physiological signalling agents. *Nat Rev Mol Cell Biol* **2020,** 21, (7), 363-383.

56. Becker, L. B., New concepts in reactive oxygen species and cardiovascular reperfusion physiology. *Cardiovasc Res* **2004,** 61, (3), 461-70.

57. Ha, T. S.; Yang, J. H.; Cho, Y. H.; Chung, C. R.; Park, C. M.; Jeon, K.; Suh, G. Y., Clinical outcomes after rescue extracorporeal cardiopulmonary resuscitation for out-of-hospital cardiac arrest. *Emerg Med J* **2017,** 34, (2), 107-111.

58. Kim, Y. J.; Ahn, S.; Sohn, C. H.; Seo, D. W.; Lee, Y. S.; Lee, J. H.; Oh, B. J.; Lim, K. S.; Kim, W. Y., Long-term neurological outcomes in patients after out-of-hospital cardiac arrest. *Resuscitation* **2016,** 101, 1-5.

59. Friess, S. H.; Sutton, R. M.; French, B.; Bhalala, U.; Maltese, M. R.; Naim, M. Y.; Bratinov, G.; Arciniegas Rodriguez, S.; Weiland, T. R.; Garuccio, M.; Nadkarni, V. M.; Becker, L. B.; Berg, R. A., Hemodynamic directed CPR improves cerebral perfusion pressure and brain tissue oxygenation. *Resuscitation* **2014,** 85, (9), 1298-303.

60. Hayes, M. M.; Berg, R. A.; Otto, C. W., Monitoring during cardiac arrest: are we there yet? *Curr Opin Crit Care* **2003,** 9, (3), 211-7.

61. Kleinman, M. E.; Perkins, G. D.; Bhanji, F.; Billi, J. E.; Bray, J. E.; Callaway, C. W.; de Caen, A.; Finn, J. C.; Hazinski, M. F.; Lim, S. H.; Maconochie, I.; Morley, P.; Nadkarni, V.; Neumar, R. W.; Nikolaou, N.; Nolan, J. P.; Reis, A.; Sierra, A. F.; Singletary, E. M.; Soar, J.; Stanton, D.; Travers, A.; Welsford, M.; Zideman, D., ILCOR Scientific Knowledge Gaps and Clinical Research Priorities for Cardiopulmonary Resuscitation and Emergency Cardiovascular Care: A Consensus Statement. *Resuscitation* **2018,** 127, 132-146.

62. Maciel, C. B.; Barden, M. M.; Greer, D. M., Neurologic Recovery After Cardiac Arrest: a Multifaceted Puzzle Requiring Comprehensive Coordinated Care. *Curr Treat Options Cardiovasc Med* **2017,** 19, (7), 52.

63. Sutton, R. M.; Friess, S. H.; Naim, M. Y.; Lampe, J. W.; Bratinov, G.; Weiland, T. R., 3rd; Garuccio, M.; Nadkarni, V. M.; Becker, L. B.; Berg, R. A., Patient-centric blood pressure-targeted cardiopulmonary resuscitation improves survival from cardiac arrest. *Am J Respir Crit Care Med* **2014,** 190, (11), 1255-62.

64. Nunes, L. B.; Mendes, P. V.; Hirota, A. S.; Barbosa, E. V.; Maciel, A. T.; Schettino, G. P.; Costa, E. L.; Azevedo, L. C.; Park, M., Severe hypoxemia during veno-venous extracorporeal membrane oxygenation: exploring the limits of extracorporeal respiratory support. *Clinics (Sao Paulo)* **2014,** 69, (3), 173-8.

65. Beyea, M. M.; Tillmann, B. W.; Iansavichene, A. E.; Randhawa, V. K.; Van Aarsen, K.; Nagpal, A. D., Neurologic outcomes after extracorporeal membrane oxygenation assisted CPR for resuscitation of out-of-hospital cardiac arrest patients: A systematic review. *Resuscitation* **2018,** 130, 146-158.

66. Han, K. S.; Kim, S. J.; Lee, E. J.; Jung, J. S.; Park, J. H.; Lee, S. W., Experience of extracorporeal cardiopulmonary resuscitation in a refractory cardiac arrest patient at the emergency department. *Clin Cardiol* **2019,** 42, (4), 459-466.

67. Ortega-Deballon, I.; Hornby, L.; Shemie, S. D.; Bhanji, F.; Guadagno, E., Extracorporeal resuscitation for refractory out-of-hospital cardiac arrest in adults: A systematic review of international practices and outcomes. *Resuscitation* **2016,** 101, 12-20.

68. Brooks, S. C.; Anderson, M. L.; Bruder, E.; Daya, M. R.; Gaffney, A.; Otto, C. W.; Singer, A. J.; Thiagarajan, R. R.; Travers, A. H., Part 6: Alternative Techniques and Ancillary Devices for Cardiopulmonary Resuscitation: 2015 American Heart Association Guidelines Update for Cardiopulmonary Resuscitation and Emergency Cardiovascular Care. *Circulation* **2015,** 132, (18 Suppl 2), S436-43.

69. Garcia-Dorado, D.; Rodriguez-Sinovas, A.; Ruiz-Meana, M.; Inserte, J.; Agulló, L.; Cabestrero, A., The end-effectors of preconditioning protection against myocardial cell death secondary to ischemia-reperfusion. *Cardiovasc Res* **2006,** 70, (2), 274-85.

70. Beyersdorf, F.; Unger, A.; Wildhirt, A.; Kretzer, U.; Deutschländer, N.; Krüger, S.; Matheis, G.; Hanselmann, A.; Zimmer, G.; Satter, P., Studies of reperfusion injury in skeletal muscle: preserved cellular viability after extended periods of warm ischemia. *J Cardiovasc Surg (Torino)* **1991,** 32, (5), 664-76.

71. Buckberg, G. D., STUDIES OF CONTROLLED REPERFUSION AFTER ISCHEMIA: I. When is cardiac muscle damaged irreversibly? *The Journal of Thoracic and Cardiovascular Surgery* **1986,** 92, (3), 483-487.

72. Haab, F.; Julia, P.; Nochy, D.; Cambillau, M.; Fabiani, J. N.; Thibault, P., Improvement of postischemic renal function by limitation of initial reperfusion pressure. *J Urol* **1996,** 155, (3), 1089-93.

73. Halldorsson, A. O.; Kronon, M.; Allen, B. S.; Rahman, S.; Wang, T.; Layland, M.; Sidle, D., Controlled reperfusion prevents pulmonary injury after 24 hours of lung preservation. *Ann Thorac Surg* **1998,** 66, (3), 877-84; discussion 884-5.

74. Allen, B. S.; Ko, Y.; Buckberg, G. D.; Tan, Z., Studies of isolated global brain ischaemia: III. Influence of pulsatile flow during cerebral perfusion and its link to consistent full neurological recovery with controlled reperfusion following 30 min of global brain ischaemia. *Eur J Cardiothorac Surg* **2012,** 41, (5), 1155-63.

75. Allen, B. S.; Ko, Y.; Buckberg, G. D.; Tan, Z., Studies of isolated global brain ischaemia: II. Controlled reperfusion provides complete neurologic recovery following 30 min of warm ischaemia - the importance of perfusion pressure. *Eur J Cardiothorac Surg* **2012,** 41, (5), 1147-54.

76. Foerster, K.; Benk, C.; Beyersdorf, F.; Cristina Schmitz, H.; Wittmann, K.; Taunyane, I.; Heilmann, C.; Trummer, G., Twenty minutes of normothermic cardiac arrest in a pig model: the role of short-term hypothermia for neurological outcome. *Perfusion* **2018,** 33, (4), 270-277.

77. Foerster, K.; D'Inka, M.; Beyersdorf, F.; Benk, C.; Nguyen-Thanh, T.; Mader, I.; Fritsch, B.; Ihling, C.; Mueller, K.; Heilmann, C.; Trummer, G., Prolonged cardiac arrest and resuscitation by extracorporeal life support: favourable outcome without preceding anticoagulation in an experimental setting. *Perfusion* **2013,** 28, (6), 520-8.

78. Taunyane, I. C.; Benk, C.; Beyersdorf, F.; Foerster, K.; Cristina Schmitz, H.; Wittmann, K.; Mader, I.; Doostkam, S.; Heilmann, C.; Trummer, G., Preserved brain morphology after controlled

automated reperfusion of the whole body following normothermic circulatory arrest time of up to 20 minutes. *Eur J Cardiothorac Surg* **2016,** 50, (6), 1025-1034.

79. Trummer, G.; Foerster, K.; Buckberg, G. D.; Benk, C.; Heilmann, C.; Mader, I.; Feuerhake, F.; Liakopoulos, O.; Brehm, K.; Beyersdorf, F., Successful resuscitation after prolonged periods of cardiac arrest: a new field in cardiac surgery. *J Thorac Cardiovasc Surg* **2010,** 139, (5), 1325-32, 1332.e1-2.

80. Trummer, G.; Foerster, K.; Buckberg, G. D.; Benk, C.; Mader, I.; Heilmann, C.; Liakopoulos, O.; Beyersdorf, F., Superior neurologic recovery after 15 minutes of normothermic cardiac arrest using an extracorporeal life support system for optimized blood pressure and flow. *Perfusion* **2014,** 29, (2), 130-8.

81. Allen, B. S.; Castellá, M.; Buckberg, G. D.; Tan, Z., Conditioned blood reperfusion markedly enhances neurologic recovery after prolonged cerebral ischemia. *J Thorac Cardiovasc Surg* **2003,** 126, (6), 1851-8.

82. Stub, D.; Bernard, S.; Duffy, S. J.; Kaye, D. M., Post cardiac arrest syndrome: a review of therapeutic strategies. *Circulation* **2011,** 123, (13), 1428-35.
